# Cost-effectiveness of introducing national seasonal influenza vaccination for adults aged 60 years and above in mainland China: a modelling analysis

**DOI:** 10.1186/s12916-020-01545-6

**Published:** 2020-04-14

**Authors:** Juan Yang, Katherine E. Atkins, Luzhao Feng, Marc Baguelin, Peng Wu, Han Yan, Eric H. Y. Lau, Joseph T. Wu, Yang Liu, Benjamin J. Cowling, Mark Jit, Hongjie Yu

**Affiliations:** 1 School of Public Health, Fudan University, Key Laboratory of Public Health Safety, Ministry of Education, Shanghai, China; 2 Centre for Mathematical Modelling of Infectious Diseases, London School of Hygiene and Tropical Medicine, London, UK; 3 Department of Infectious Disease Epidemiology, London School of Hygiene & Tropical Medicine, London, UK; 4 Centre for Global Health Research, Usher Institute of Population Health Sciences and Informatics, The University of Edinburgh, Edinburgh, UK; 5 Key Laboratory of Surveillance and Early-warning on Infectious Disease, Division of Infectious Disease, Chinese Center for Disease Control and Prevention, Beijing, China; 6 MRC Centre for Global Infectious Disease Analysis, School of Public Health, Imperial College London, London, UK; 7 WHO Collaborating Centre for Infectious Disease Epidemiology and Control, School of Public Health, Li Ka Shing Faculty of Medicine, The University of Hong Kong, Hong Kong Special Administrative Region, China; 8 Modelling and Economics Unit, Public Health England, London, UK

**Keywords:** Influenza, Older adults, Vaccination, China, Cost-effectiveness analysis

## Abstract

**Background:**

China has an aging population with an increasing number of adults aged ≥ 60 years. Influenza causes a heavy disease burden in older adults, but can be alleviated by vaccination. We assessed the cost-effectiveness of a potential government-funded seasonal influenza vaccination program in older adults in China.

**Methods:**

We characterized the health and economic impact of a fully funded influenza vaccination program for older adults using China-specific influenza disease burden, and related cost data, etc. Using a decision tree model, we calculated the incremental costs per quality-adjusted life year (QALY) gained of vaccination from the societal perspective, at a willingness-to-pay threshold equivalent to GDP per capita (US$8840). Moreover, we estimated the threshold vaccination costs, under which the fully funded vaccination program is cost-effective using GDP per capita as the willingness-to-pay threshold.

**Results:**

Compared to current self-paid vaccination, a fully funded vaccination program is expected to prevent 19,812 (95% uncertainty interval, 7150–35,783) influenza-like-illness outpatient consultations per year, 9418 (3386–17,068) severe acute respiratory infection hospitalizations per year, and 8800 (5300–11,667) respiratory excess deaths due to influenza per year, and gain 70,212 (42,106–93,635) QALYs per year. Nationally, the incremental costs per QALY gained of the vaccination program is US$4832 (3460–8307), with a 98% probability of being cost-effective. The threshold vaccination cost is US$10.19 (6.08–13.65). However, variations exist between geographical regions, with Northeast and Central China having lower probabilities of cost-effectiveness.

**Conclusions:**

Our results support the implementation of a government fully funded older adult vaccination program in China. The regional analysis provides results across settings that may be relevant to other countries with similar disease burden and economic status, especially for low- and middle-income countries where such analysis is limited.

**Electronic supplementary material:**

**Supplementary information** accompanies this paper at 10.1186/s12916-020-01545-6.

## Background

Seasonal influenza is a major cause of mortality, with recent estimates suggesting that 291,000–646,000 influenza-associated respiratory deaths occur globally each year [1]. Older adults are at increased risk of hospitalization or death if infected and thus are included in the recommended groups for annual influenza vaccination by the World Health Organization (WHO) [2]. The World Health Assembly set a target of attaining vaccination coverage of 75% in this group by 2010 [3]. Most high-income countries and many upper middle-income countries, like Thailand and Brazil, have incorporated seasonal influenza vaccination for older adults into their National Immunization Program, which has significantly increased vaccination uptake [4–6].

As the world’s most populous country, China has more adults ≥ 60 years (> 210 million in 2016) than any other country, accounting for nearly a quarter of the global total. China is also aging rapidly; adults ≥ 60 years account for 15% of the population in 2016 [7] and will increase to 26% by 2030 [8]. Influenza caused 66–105 severe acute respiratory infection (SARI) hospitalizations per 100,000 adults ≥ 60 years in China [9, 10]. Annually, over 80% of influenza-related excess deaths occurred in older adults [11, 12], with an average excess respiratory mortality rate per season estimated at 38.5 (95% confidence interval, 95%CI 36.8–40.2) per 100,000 persons between 2010 and 2015 [12]. However, there is no nationwide government-funded influenza vaccination program for older adults in China, and the cost of vaccination is completely borne by individuals. This self-paid vaccination system contributes to an extremely low vaccine uptake of 4% in this age group, far behind the target of 75% [13]. Only a handful of relatively wealthy cities provide free influenza vaccination for older adults paid by local governments [14]. For example, since 2007, Beijing has provided free influenza vaccination to older adults, leading to the uptake reaching 39% in 2012 [15].

Following a health scare involving improper refrigeration of transported vaccines sold privately nationwide in 2016 [16], the State Council of China recommended acceleration of the inclusion into the National Immunization Program of vaccines currently sold in the private sector [17]. The new vaccine administration law in 2019 requires establishing a “national dynamic adjustment mechanism” for inclusion/exclusion of vaccines into National Immunization Program [18]. Both the State Council and National Immunization Advisory Committee also recommended taking into consideration the cost-effectiveness of vaccination alongside traditional considerations of vaccine efficacy and safety for vaccine policy-making [18].

A systematic review of cost-effectiveness studies of influenza vaccination showed that globally a third of studies (8/27) found vaccination in older adults to be cost-saving, and most of the remainder found vaccination to be cost-effective [19]. However, to date no comprehensive study has been conducted in mainland China, where the economic impact of fully funded vaccination programs may differ greatly across regions due to large variations in influenza seasonality, disease burden, demographic structure, and social economic development [11, 20, 21]. Hence, the objective of this study is to answer the question of whether a fully funded influenza vaccination program for nearly a quarter of the world’s older adult population is an efficient use of resources in mainland China, and to further explore whether variations in this result exist across geographical regions.

## Methods

Following WHO guidance on the economic evaluation of influenza vaccination [22], we performed a cost-effectiveness analysis of a government-funded influenza vaccination program for adults ≥ 60 years compared to the status quo of vaccinees paying out-of-pocket (hereafter “fully funded vaccination program” and “self-paid vaccination program” respectively) from the societal perspectives. As most costs and effects due to influenza occur during a single influenza season, we used a time horizon of 1 year, with the exception of tracking all the years of life lost when a patient died of influenza-related causes.

### Decision tree model

**Fig. 1:**
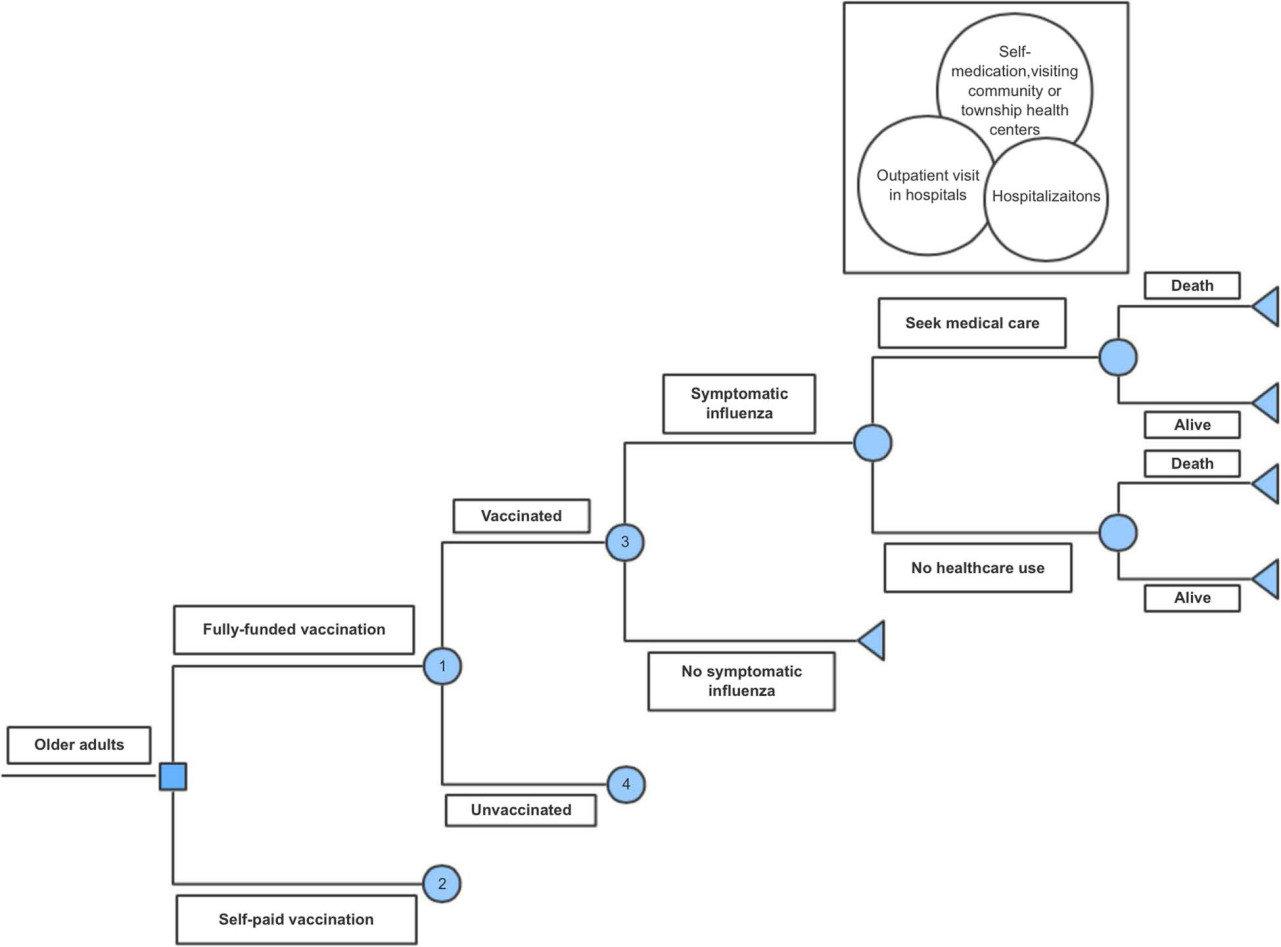
Decision tree model for influenza vaccination in older adults. Chance node 2 is the same as chance node 1, and chance node 4 is the same as chance node 3

We developed a static decision tree model (Fig. 1) to calculate the per person costs of vaccination, per person costs due to influenza, and per person health utility loss due to influenza. From these estimates, we estimated the impact of the fully funded program compared to self-paid vaccination on health and economic outcomes at the regional and national level. We then used these outcomes to calculate the incremental cost-effectiveness of the fully funded program. Detailed methods are shown in Additional file 1.

As current vaccine coverage is only 4% and is concentrated in a few highly developed cities with local government funding [13], we assumed the probability of being vaccinated was zero under the status quo. There is significant uncertainty in the vaccine uptake that may be achieved in a potential fully funded vaccination program. The experience of Beijing showed that the uptake in older adults increased substantially from 2% in 1999 to 39% in 2012 [15, 23] after fully funded influenza vaccination was offered in 2007. It is likely that the uptake in other less densely populated and developed provinces would not increase as quickly as Beijing, the capital of China, where residents likely to have greater access to health care facilities. We therefore used a conservative coverage assumption of 30% in the analysis.

An older adult is assumed to have a risk of acquiring a symptomatic influenza infection annually. Someone with symptomatic influenza then has a probability of seeking medical treatment, including self-medication, seeking healthcare in a community or township health service center, consulting a doctor in an outpatient department, or being hospitalized. Each infected person also has a probability of dying of influenza-related causes, whether or not the person has received healthcare.

The models were stratified by area (rural/urban) and geographical regions (Additional file 2: Figure S1: Northern, Northeast, Northwest, Eastern, Central, Southwest, and Southern). All analyses were performed in R version 3.5.0 (https://www.r-project.org).

### Data sources

#### Population

The model tracked older adults aged 60–64, 65–69, 70–74, 75–79, and ≥ 80 years. The age-specific population size in 2016 was obtained from the National Bureau of Statistics in China and stratified by area (rural/urban) using the proportion of older persons living in urban areas reported in the 2010 Population Census of China [24] (Additional file 3: Table S1).

Older adults were further split into high- and low-risk groups. High-risk individuals are defined as those with an increased risk of hospitalization or death if infected by influenza due to underlying medical conditions as listed in the WHO influenza vaccine guidelines, including chronic obstructive pulmonary disease, asthma, diabetes, and chronic cardiac disease [25] The remaining population was categorized as low risk. The probability of an older adult having at least one underlying medical disease was estimated from the results of the China Health and Retirement Longitudinal Study [26, 27], a nationally representative study on health status in older people (Additional file 4: Figures S2–S3).

### Influenza-related disease burden

#### Influenza-like-illness (ILI) consultations due to influenza

The yearly average risk of ILI-related primary care or outpatient consultations due to influenza in China was estimated to be 0.9 per 1000 (95% CI 0.4–1.5) between 2010 and 2015 [28]. The influenza-related ILI consultation risk varied significantly cross provinces (Additional file 5: Table S2), ranging from 10 to 690 per 100,000.

#### Hospitalization

It was found that influenza was associated with an estimated 89 (95%CI 85–90) SARI hospitalizations per 100,000 for individuals ≥ 65 years during 2011–2012 in Jingzhou (a city in Southern China) [9]. The rates were 105 (95%CI 85–129) and 66 (95%CI 50–86) per 100,000 people in Beijing (a province in Northern China) during the 2014–2015 and 2015–2016 seasons, respectively [10]. In our study, the influenza-related hospitalization rates in other Southern and Northern provinces (Additional file 2: Figure S1) were estimated using the local influenza-related ILI consultation rate multiplied by the ratio of influenza-related SARI hospitalization rate to influenza-related ILI consultation rate separately in Jingzhou and Beijing [9, 10, 28].

#### Mortality

The national average influenza-associated excess mortality attributable to respiratory diseases was estimated to be 38.5 (95%CI 36.8–40.2) per 100,000 between 2010 and 2015 in China [12]. Variation (19.0–83.2/100,000) was observed across provinces (Additional file 5: Table S2).

We found a clear positive relationship between Gross Regional Product per capita and influenza-related ILI consultation risk (Pearson correlation coefficient = 0.83, *p* < 0.05). This variation is likely to be explained by differences in health care access or under-reporting. In the base case analysis, we used original influenza-related ILI consultation and excess mortality rates as reported for each province in the literatures [12, 28]. This assumes that the differences between provinces are genuine and are explained by differences in influenza epidemiology.

The highest influenza-related ILI consultation risk occurs in Shanghai (690/100,000), a high-income province with very good health care access and surveillance system. Accordingly, in the scenario analyses, we assumed every province has the same risk as Shanghai based on the “under-reporting” hypothesis or assumed the differences are explained by differences in health care access (i.e., “health care access” hypothesis).

For excess mortality, we assumed every province has the same risk as the province with the highest risk, which is 83.2/100,000 in Gansu province [12]. A total of four scenario analyses were performed in this study, with detailed descriptions shown in Table 1.

**Table 1:** Description of base case and scenario analyses

Analyses	Influenza-related ILI consultation [28]	Influenza-associated excess mortality attributable to respiratory diseases [12]
Base case	Used original rate as reported for each province in the literature	Used original rate as reported for each province in the literature
Scenario 1	Used original rate as reported for each province in the literature, and assumed the difference between any other province and Shanghai is due to difference in health care access (i.e., “health care access” hypothesis)	Assumed every province has the same risk as Gansu Province, with the highest rate of 83.2/100,000
Scenario 2	Assumed every province has the same risk as Shanghai, with the highest rate of 690/100,000	Assumed every province has the same risk as Gansu Province, with the highest rate of 83.2/100,000
Scenario 3	Used original rate as reported for each province in the literature, and assumed the difference between any other province and Shanghai is due to difference in health care access (i.e., “health care access” hypothesis)	Used original rate as reported for each province in the literature
Scenario 4	Assumed every province has the same risk as Shanghai, with the highest rate of 690/100,000	Used original rate as reported for each province in the literature

A systematic review demonstrated that the presence of “any risk factor” (using the WHO risk factors definition [25]) was associated with an increased risk of hospital admission (odds ratio 3.39, 95%CI 2.60–4.42) and death (odds ratio 2.04, 95%CI 1.74–2.39) in influenza-related patients [29].

### Healthcare seeking behavior

A household survey on health seeking behavior of adult patients with acute respiratory infections carried out in China during 11/2009–03/2010, found that (1) in urban areas, 9.7% of acute respiratory infection cases did not seek any medical help, 66.0% self-medicated, or visited a doctor in community or township health centers, and the remaining 24.3% visited a doctor in county or higher-level hospitals; (2) in rural areas, the relevant proportions were respectively 8.6%, 79.0%, and 12.4% [30]. We assumed that influenza patients have the same healthcare-seeking behaviors as acute respiratory infections cases.

### Influenza-related costs

We used the average drug cost per outpatient in township healthcare centers (US$ 5.4 in 2017) and that in community healthcare centers (US$11.9 in 2017) as a proxy of the cost for self-medication of influenza patients in urban and rural areas, respectively [31]. We previously found the treatment costs for influenza-related outpatients and inpatients aged 60 years old and over were respectively US$129 (95% uncertainty interval, 95%UI 75–156) and US$2735 (1401-4482) in East China in 2013 [32]. The costs were extrapolated to other regions in China in proportion to the regional GDP (gross domestic product) per capita.

We also considered the lost productivity due to premature mortality attributable to influenza, which was estimated using the friction cost method. The length of the friction period was assumed to be 3 months, the elasticity of labor time versus production assumed to be 0.8, and the costs of filling a vacancy and training new personnel estimated to be US$357 in 2009 [33, 34]. The yearly income per capita of older adults (urban US$3896; rural US$1241) was obtained from the fourth survey of the living conditions of older adults in urban/rural China in 2014 [35]. The labor force participation rates of older adults were derived from the 2010 Population Census of China [24]. All costs were adjusted and converted to US dollars in 2017 using the consumer price index and the exchange rate of 1 US$ = 6.75 CNY [36].

### Quality-adjusted life years (QALYs) lost

The number of QALYs lost due to influenza was calculated as the sum of QALYs lost due to non-fatal episodes plus life years lost due to fatal episodes. The duration of non-fatal episodes was assumed to be respectively 6.2 days (standard deviation, 2.2) and 16.0 days (10.7) for influenza-related outpatients and inpatients. Their associated health utility was separately estimated to be 0.5733 (95%UI 0.4650–0.6608) and 0.4128 (0.1793–0.6380) [37]. The background health utility (urban 0.7719–0.8071; rural 0.6943–0.7434) was obtained from the China Health and Retirement Longitudinal Study [38].

Life years lost due to fatal episodes were estimated based on risk-, area-, and age-specific life expectancy. Life expectancy was calculated using the life table approach and mortality data in 2017 from China Health and Family Planning Statistical Yearbook [31, 39]. Life years lost were discounted at an annual rate of 3% [40] (Additional file 6: Tables S3–S4).

### Vaccine effectiveness and cost

Unlike the US Flu Vaccine Effectiveness network [41], there is no regular evaluation of influenza vaccine effectiveness in mainland China. Even though a few studies have been conducted to try to evaluate the local influenza vaccine effectiveness in the older adults in the Capital city Beijing during 2013–2016 [42, 43], the estimates were not precise due to limited sample size, and it cannot represent the effectiveness across China due to large variations in terms of seasonality and activity of influenza virus [20].

A recent meta-analysis of test-negative design case-control studies conducted between 2004 and 2013 indicated that influenza vaccine is effective against laboratory-confirmed influenza (odds ratio 0.48; 95% CI 0.39–0.59) in older adults when the vaccine strains closely match the circulating influenza viruses, and also had significant effectiveness when vaccine is poorly matched (odds ratio 0.64; 95% CI 0.52–0.78) [44]. We conservatively used the efficacy of poorly matched vaccines in the baseline analysis. Adverse effects associated with influenza vaccination which were not considered as serious adverse events are extremely rare [45].

The procurement cost of influenza vaccination (not including vaccine logistic and administration costs) in 2013 was US$5.73 per dose (95%UI 5.43–6.03) for the 0.50 ml formulation trivalent inactivated influenza vaccine [14].

### Socioeconomic status

Luo and Xie [46] found that 74.5% of older adults were economically independent (i.e., their daily expenses could be paid by their retirement wage/pension or other income) in China. They also found older adults with economic independence had 26.4% lower risk of respiratory diseases mortality. Hence, we further calculated the benefits and cost-effectiveness of vaccination by economic independence.

### Outcome measures

In this study, we calculated the incremental costs per QALY gained of vaccination and evaluated the health and economic impact of fully funded influenza vaccination at the national and seven regional levels, respectively. Because China does not have an official threshold for cost-effectiveness, we used a willingness-to-pay threshold of the GDP per capita (US$8840 in 2017) in the base case analysis, and a more stringent threshold of US$3780–US$5880 per QALY gained proposed by University of York economists [47] to construct cost-effectiveness acceptability curves (CEAC). Due to the unavailability of vaccine logistic and administration costs, only vaccine procurement costs were included in the base case and sensitivity analyses. We further performed analyses for threshold vaccination costs (TVC), below which fully funded vaccination program would be considered cost-effective.

### Sensitivity analysis

We performed probabilistic sensitivity analyses to explore the influence of all parameters on incremental cost-effectiveness ratios (ICERs). This was done using Monte Carlo sampling with applicable distributions for different parameters (Table 2), drawing 10,000 samples, then calculating the median, and 95% UIs for the ICERs based on the 2.5th and 97.5th percentiles of the 10,000 simulations. Scenario sensitivity analyses were also conducted (1) from the health system perspective (only considering the direct medical costs for influenza patients), (2) using well-matched vaccine effectiveness [44], (3) using a discount rate of zero for QALYs loss as recommended by WHO guidelines [48], and (4) considering the circulating influenza virus strains change after 2009 H1N1 pandemic, the most recent vaccine effectiveness between 2013 and 2018 in the USA (range 12–50%) [49] were used in the sensitivity analysis in order to evaluate its impact on our outcomes, with the vaccine effectiveness of 12% and 50% separately for mismatched and well-matched vaccines.

**Table 2:** Key model parameter distributions

Parameter	Mean (range/standard deviation)*	Distribution
Proportion of high-risk groups	Additional file 4: Fig. S3	Beta
Flu-related ILI consultation rate [28]	Additional file 5: Table S2	Normal
Flu-related SARI hospitalization (per 100,000) [9]		
Beijing (2013–2014)	105 (95%CI 85–129)	Normal with *μ* = 105, sd = 11.22
Beijing (2014–2015)	66 (95%CI 50–86)	Normal with *μ* = 66, sd = 9.18
Jingzhou, Hubei province (2011–2012)	89 (95%CI 85–90)	Uniform (min = 85/100,000, max = 90/100,000)
Flu-related respiratory excess mortality [12]	Additional file 5: Table S2	Lognormal
Healthcare-seeking behavior (%) [30]		
Probability of no-healthcare-use	Urban 9.7,	Urban: Dirichlet with α1 = 107, α2 = 704, α3 = 269, Rural: Dirichlet with α1 = 43, α2 = 394, α3 = 62
	Rural 8.6	
Probability of self-treatment, seeking care in Community/Township Health Service Centers	Urban 66.0,	
	Rural 79.0	
Probability of visiting doctors in county-level and above hospitals	Urban 24.3	
	Rural 12.4	
Odds ratio of influenza-related hospitalization in high-risk groups compared to low-risk groups [29]	3.39	Lognormal with *μ* = 1.22, sd = 0.14
Odds ratio of influenza-related death in high-risk groups compared to low-risk groups [29]	2.04	Lognormal with *μ* = 0.71, sd = 0.08
Vaccine cost (US$ in 2013) [14]	5.73 (95%UI 5.43–6.03)	Bootstrap from data on influenza vaccine cost
Influenza outpatients visits and hospitalization costs (US$ in 2013) [32]	Outpatients: 129 (95%UI 75–156)	Bootstrap from data on national retrospective survey
	Inpatients: 2735 (95%UI 1401–4482)	
Duration of influenza episode for outpatients and inpatients (days) [37]	Outpatients: 6.2 (SD 2.2)	Bootstrap from data on national retrospective survey
	Inpatients: 16.0 (SD 10.7)	
Utility of influenza outpatients and inpatients [37]	Outpatients: 0.5733 (95%UI 0.4650–0.6608)	Bootstrap from data on national retrospective survey
	Inpatients: 0.4128 (95%UI 0.1793–0.6380)	
Background health utility [38]	Urban 60–74 years: 0.8071 (SD 0.0039); ≥ 75 years: 0.7719 (SD 0.0093) Rural 60–74 years: 0.7434 (SD 0.0031); ≥ 75 years: 0.6943 (SD 0.0078)	Normal distribution
Risk of infected from influenza in vaccinated group vs. unvaccinated group (odds ratio) [44]	0.64 (0.52–0.78)	Lognormal with *μ* = − 0.45, sd = 0.10

*Used in one-way sensitivity analysis. 95%CI denotes 95% confidence interval; 95%UI denotes 95% uncertainty interval calculated by bootstrap methods

## Results

### Impact and cost-effectiveness in the base case scenario

**Fig. 2:**
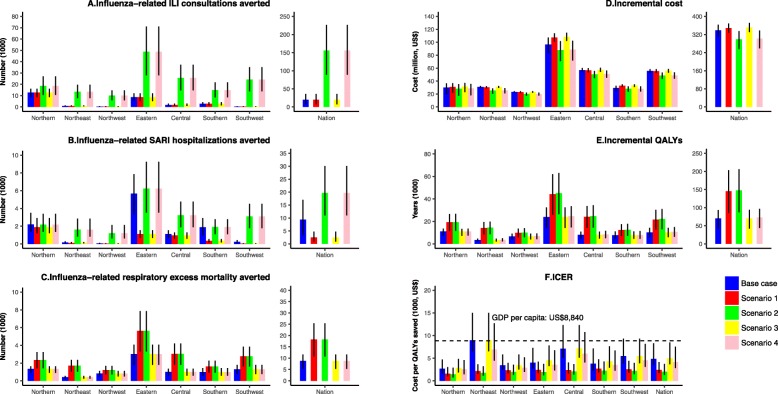
**a**–**f** Epidemiological and economic impact of fully funded influenza vaccination program in older adults, stratified by geographic regions, China

At the national level, a total of 63.4 million older adults in China are expected to be vaccinated annually. Vaccination is expected to prevent 19,812 (95%UI 7150–35,783) influenza-related ILI outpatient consultations per year, 9418 (3386–17,068) influenza-related SARI hospitalizations per year, and 8800 (5300–11,667) influenza-related deaths due to respiratory diseases per year, with separately 40%, 69%, and 57% occurring in high-risk groups (Fig. 2 and Additional file 7: Figure S4).

**Fig. 3:**
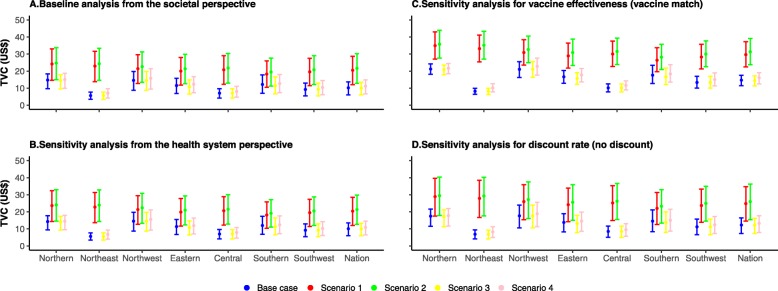
**a**–**d** Threshold vaccination costs (TVC)

The fully funded vaccination program is estimated to cost US$ 339 (310–363) million, but gain 70,212 (42,106-93,635) QALYs, 98% of which were due to influenza-related excess deaths averted (Fig. 2). A total of 38% of the increment cost and 54% of incremental QALYs occur in high-risk groups (Additional file 7: Figure S4). The QALYs gained by vaccination were 3 per 10,000 persons at an incremental cost of US$16,111 per 10,000 persons in older adults with economic independence and 4 per 10,000 persons at an incremental cost of US$15,763 per 10,000 persons in older adults without economic independence. Using the GDP per capita as a threshold, the fully funded vaccination in older adults in China is cost-effective with an ICER of US$4832 (3460-8307) per QALY gained. The TVC is US$10.19 (6.08–13.65), under which the fully funded vaccination program is cost-effective using GDP per capita as the willingness-to-pay threshold (Fig. 3).

Substantial variations in health and economic outcomes are observed across regions (Fig. 2). Except in Northeast China (US$8945), the median ICER (US$2691–7115) is below the GDP per capita and hence cost-effective. The TVC in Northeast and Central China is lower than the national average, decreasing to US$5.66 (3.41–7.70) and US$7.06 (4.15–9.66) (Fig. 3).

### Probabilistic sensitivity analyses

**Fig. 4:**
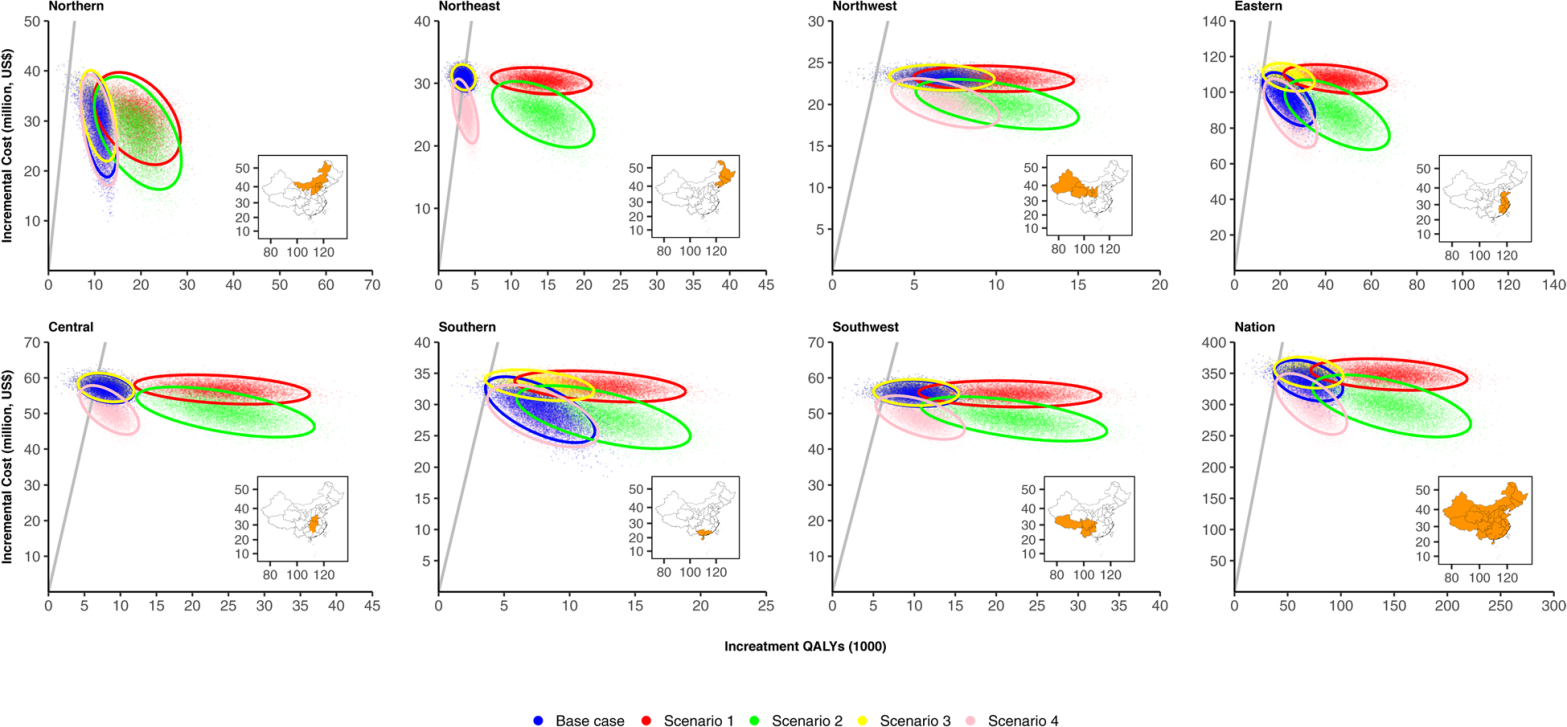
Monte Carlo simulation results on the cost-effectiveness for fully funded vaccination program compared to self-paid vaccination program (gray line denotes China’s GDP per capita in 2017 and circle denotes the 95%UI)

**Fig. 5:**
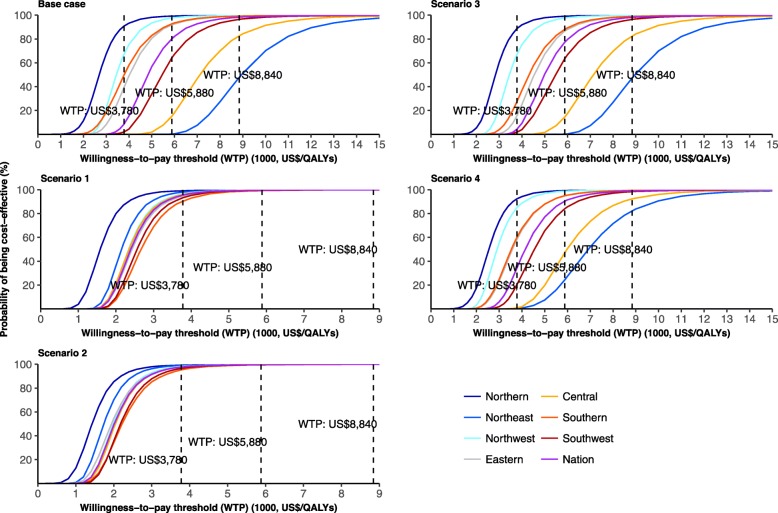
Cost-effectiveness acceptability curve (US$3780 and US$5880 denote the willingness-to-pay thresholds calculated by Ochalek [46], while US$8840 is the GDP per capita in 2017, China)

At the national level, 98% of Monte Carlo samples are considered cost-effective under base case assumptions with a threshold of GDP per capita. However, significant differences are observed for regions. For Northeast and Central China, the proportion respectively reduces to 48% and 82%. While for other regions, the probability is over 96% (Fig. 4). Using a much more stringent threshold of US$3780–5880 per QALY gained [47], the probability of cost-effective for vaccination decreases to 9–80% at the national level. Similar patterns are observed across regions (Fig. 5). At the national level, the probability of cost-effectiveness of vaccination was 99% in high-risk groups and 86% in low-risk groups (Additional file 7: Figure S5); 97% for older adults with economic independence, and 99% for those without economic independence.

### Scenario analyses

Compared to the base case scenario, the influenza-related excess mortality due to respiratory diseases increased 1–3 folds in Central, Northeast, and Southwest China, while only 47–86% for other regions in scenarios 1 and 2. The low probability of being cost-effective (around 48%) is only observed for Northeast China in base case and scenario 2 (Fig. 4). Compared to the base case scenario, TVC increases by 55–330% in scenarios 1 and 2, and 4–24% in scenario 4, while decreases slightly in scenario 3 (Fig. 3).

Compared to the base case scenario, for scenarios 1 and 2, the probabilities of being cost-effective are much higher; they are over 80% across regions even using a much more stringent threshold of US$3780 per QALY gained [47], and all are 100% using a willingness-to-pay threshold of GDP per capita. Similar patterns as the base case are observed for scenarios 3 and 4 (Fig. 5).

Compared to the societal perspective analysis above, ICERs increase slightly (mostly by < 10% depending on region and scenario) from a healthcare provider perspective (Additional file 8: Figures S6–S8). Vaccine effectiveness and discount rate have a high impact on ICERs. When the vaccine is well-matched circulating influenza strains, the fully funded vaccination program is 100% cost-effective across all regions (Additional file 9: Figures S9–S11). When the discount rate for QALYs loss is zero, the fully funded vaccination program is cost-effective across all regions, at a probability of > 90% except for Northern China in base case and scenario 3 (around 80%) (Additional file 10: Figures S12–S14).

Compared to the base case analysis (with a mismatched vaccine and a discount rate of 3%) from the societal perspective, the TVC decreases by less than 5% from the healthcare provider perspective, while it increases by 44–45% when vaccine strains match the circulating strains, and increases by 18–21% when discount rate is zero for QALYs loss (Fig. 3).

Compared to using a vaccine effectiveness of 36% in the baseline analysis [44], using an vaccine effectiveness of 12% in USA (mismatched) [49] resulted in a decrease of around 67% in terms of national influenza-related ILI consultations, hospitalizations, and deaths averted and QALYs gained separately, while an increase of 5% in incremental costs, leading to median ICER (US$ 15,190) beyond GDP per capita for base case scenario. The probability of being cost-effective for scenarios 1 and 2 remained at 99–100%. Using a vaccine effectiveness of 50% in the USA (matched) [49], national influenza-related ILI consultations, hospitalizations, and deaths averted and QALYs gained increased by 39–44%, while incremental cost decreased by 1–9%, leading to 100% probability of being cost-effective for all scenarios (details shown in the Additional file 11: Table S5). Similar patterns are observed across geographic regions.

## Discussion

The provision and management of vaccines in China is currently undergoing regulatory reforms [16, 50]. Expanding China’s government-funded vaccination programs is now recommended by both WHO and the State Council of China [17, 51]. In 2019, the influenza vaccine was one of the vaccines that went through comprehensive evaluation by the National Immunization Advisory Committee of China for inclusion into the National Immunization Program as a fully government-funded vaccine. A first step towards this could be considering vaccination for older adults due to their higher risk of influenza-related hospitalization and mortality. Our analysis comprehensively evaluates the health and economic impact of a potential fully funded influenza vaccination program in older adults. It shows that vaccinating older adults in China is cost-effective, with an ICER of US$ 4832 per QALY gained (lower than GDP per capita), despite conservative assumptions about vaccine effectiveness assumed in the base case scenario. However, we find that variations in health and economic impact exist across regions.

In our study, the fully funded vaccination program could reduce both QALY loss and productivity loss due to premature deaths. While productivity loss only contributes to < 2% of the decrease in total costs, the relevant QALY loss averted contributes to > 96% in total QALYs saved. Accordingly, variation in influenza-related respiratory excess mortality across regions is a significant factor for different ICERs observed here (base case vs. scenarios 1 and 2). Our analysis demonstrates that in the base case analysis, the probability of being cost-effective for the fully funded influenza vaccination program is much lower in regions with lower reported mortality burden than that with heavy influenza excess mortality burden (e.g., lower in Northeast compared to. Northern China) [12]. The influenza mortality in Northeast China may genuinely be lower due to lower population density and reduced air pollution. On the other hand, it may simply appear lower due to factors such as patients seeking advanced healthcare in neighboring developed regions [12] and poor quality of influenza and death surveillance. Since the two sets of potential reasons for lower mortality are difficult to disentangle, we should be very cautious in interpreting regional-level economic results. This highlights the importance of improved influenza surveillance, particularly in less developed regions of China, in order to better target influenza control programs. Variations in influenza-related ILI consultation only have slightly impact on the ICERs (base case vs. scenario 4).

In Northeast China, the fully funded influenza vaccination program is considered cost-effective if TVC is respectively below US$5.7 in the base case analysis. We used the private sector vaccine cost in the model, which is US$5.73 per dose currently [14], much higher than most of the vaccines currently used in the National Immunization Program [52]. Several Chinese manufactures produce influenza vaccines in Northeast China [45]. A government-funded influenza vaccination program using local manufacturers’ vaccines is likely to have lower delivery costs due to economies of scale and lower procurement costs due to increased consumer bargaining power. That will certainly increase the likelihood that the fully funded influenza vaccination is cost-effective in this region.

Only two studies to date has assessed the cost-effectiveness of influenza vaccination among older adults in China [53, 54]. The study of Chen et al. [53] had a number of limitations: (i) it used influenza-related outpatient and hospitalization rates in the USA, which may not be good proxies for relevant rates in China due to the different influenza seasonality, virus activity, health-seeking behavior, etc. [20] And (ii) it used influenza-related mortality before 2009 influenza pandemic in China, even though the burden has changed due to the displacement of seasonal A(H1N1) virus after pandemic [11, 12]. With these shortcomings, the paper suggested that government-funded influenza vaccination was < 50% likely to be cost-effective, when compared to a threshold of one times GDP per capita. Similarly, the second study used the influenza-related burden in a few developed cities like Beijing and Shanghai [54]. In our study, we used the most recent China-specific post-2009 pandemic data stratified by provinces, including influenza-related outpatient, hospitalization, and mortality rates.

The number of excess respiratory deaths [28] used in this study may not fully capture all influenza-associated deaths because influenza virus infections not only cause respiratory deaths, but also deaths from other diseases such as cardiovascular diseases, diabetes, and renal diseases [11]. Accordingly, vaccination could be even more cost-effective than presented here.

In our analysis, we used the same vaccine effectiveness against laboratory-confirmed influenza-related consultation, hospitalization, and death. Castilla et al. conducted a test-negative case-control study in Spain during six influenza seasons, to compare the vaccine effectiveness in preventing laboratory-confirmed influenza in outpatient and inpatient cases at the same time and in the same population of older adults. They concluded that no difference was observed in vaccine effectiveness in general practice and hospital settings [55]. If a higher VE against hospitalizations and deaths was used in our study by assuming influenza vaccine mitigates influenza illness severity, the probability of vaccination being cost-effective would increase due to more treatment costs and productivity losses averted and more QALYs gained.

It is known that influenza burden and vaccine effectiveness vary across years partly due to predominant circulating viruses and how closely related the viruses in the vaccine are to the circulating viruses. Previously studies have demonstrated that more influenza-related consultations and greater excess mortality were observed in 2014–2015 season when influenza A(H3N2) virus predominated [28], and the effectiveness of influenza vaccines was reduced due to an antigenic mismatch between the circulating strain and vaccine virus [42]. In this case, vaccination is likely to be less cost-effective than in other years. However, in our study, we conducted the cost-effectiveness analysis using the average influenza burden after 2009 H1N1 pandemic and vaccine effectiveness across several years regardless of influenza type/subtype/lineage. This is because our work is intended to inform the decision to introduce influenza vaccination into the National Immunization Programme for the long term, rather than for a specific season or against a specific type/subtype/lineage.

A limitation of our study is that the influenza-related SARI hospitalization rate is only available in one city each in Southern and Northern China [9, 10]. These two cities may not fully represent the hospitalization rate across China. We used the ratio of the influenza-related SARI hospitalization rate to influenza-related ILI consultation rate separately in Jingzhou, Hubei, and Beijing as a multiplier to estimate the influenza-related SARI hospitalization rate for the rest of Southern and Northern China, respectively. However, it may not be a good proxy due to different health seeking behaviors especially between areas with varying levels of socioeconomic development, and health service provision.

China’s first vaccine administration law allows provincial governments to add additional vaccines into their local fully funded vaccine list on the basis of local disease burden [18]. Until now, only a few highly developed provincial- and prefecture-level cities have offered fully funded influenza vaccination for older adults (e.g., Beijing and Shenzhen). These local initiatives have achieved remarkable increases in local vaccine uptake [14, 15]. However, expanding such fully funded vaccination to the entire population or even large regions of China would require large budget allocations. Because of that, there is a need for detailed cost-effectiveness analysis to determine if such a move is good value for money. Hence, our results fill a key evidence gap needed by decision-makers in China. Due to large apparent variations in influenza disease burden, and socioeconomic development level across regions, our regional analyses could also provide information on the cost-effectiveness of fully funded influenza vaccination that may be relevant to other countries with similar disease burden and economic status, especially low- and middle-income countries where cost-effectiveness analysis is limited [19].

## Conclusions

Making use of the China-specific influenza disease burden, including influenza-related outpatient consultations, severe acute respiratory infection hospitalizations and respiratory excess deaths, as well as related cost data, we built up a decision tree model to characterize the cost-effectiveness of introducing influenza vaccination for older adults. Our results support the implementation of a government fully funded older adult vaccination program in China. The regional analysis provides results across settings that may be relevant to other low- and middle-income countries with similar disease burden and economic status.

## Supplementary information

Additional file 1: Methods of estimating the impact of vaccination program at the population level.

Additional file 2: **Figure S1.** Map showing geographical regions in mainland China.

Additional file 3: **Table S1.** Population size.

Additional file 4: **Figure S2.** Schematic diagram for estimating high-risk population size; **Figure S3.** Probability of having underlying diseases.

Additional file 5: **Table S2.** Influenza-related disease burden.

Additional file 6: **Table S3.** Age-specific mortality by rural/urban areas; **Table S4.** Life expectancy.

Additional file 7: **Figure S4.** Epidemiological and economic impact by regions and risk groups; **Figure S5.** Monte Carlo simulation results by regions and risk groups.

Additional file 8: **Figure S6.** Epidemiological and economic impact (analyses from the health system perspective); **Figure S7.** Monte Carlo simulation results (analyses from the health system perspective); **Figure S8.** CEAC (analyses from the health system perspective).

Additional file 9: **Figure S9.** Epidemiological and economic impact (analyses with well-matched vaccines); **Figure S10.** Monte Carlo simulation results (analyses with well-matched vaccines); **Figure S11.** CEAC (analyses with well-matched vaccines).

Additional file 10: **Figure S12.** Epidemiological and economic impact (analyses with a discount rate of zero for QALYs loss); **Figure S13.** Monte Carlo simulation results (analyses with a discount rate of zero for QALYs loss); **Figure S14.** CEAC (analyses with a discount rate of zero for QALYs loss).

Additional file 11: **Table S5.** Comparison of baseline analysis with that using US vaccine effectiveness.

## Data Availability

The data generating the findings of this article are included within the article and its additional files.

## Abbreviations

95%CI: 95% Confidence interval
95%UI: 95% Uncertainty interval
CEAC: Cost-effectiveness acceptability curves
GDP: Gross domestic product
ICER: Incremental cost-effectiveness ratios
ILI: Influenza-like-illness
QALYs: Quality-adjusted life years
SARI: Severe acute respiratory infections
TVC: Threshold vaccination costs
WHO: World Health Organization
