# Supplementary material for: Cost-effectiveness of introducing national seasonal influenza vaccination for adults aged 60 years and above in mainland China: a modelling analysis
Source: BMC Med. 2020 Apr 14;18:90. doi: 10.1186/s12916-020-01545-6 (PMC7155276; doi:10.1186/s12916-020-01545-6)
Supplement: Supplementary file 1 — Methods of estimating the impact of vaccination program at the population level. [file 12916_2020_1545_MOESM1_ESM.pdf]

Additional file 1. Methods of estimating the health and economic impact of government fully-funded vaccination program at the population level

Since a decision tree is a static model, the population size and the vaccination coverage does not affect the incremental cost-effectiveness ratio (ICER) between the fully-funded vaccination program and self-paid vaccination program. Accordingly, we built an individual decision tree based on the probability of being vaccinated to figure out the individual health and economic outcomes (including health utility due to influenza per person, and cost of vaccination and cost due to influenza per person) separately for the government fully-funded vaccination program and self-paid vaccination program at the first step. Secondly, ICER was calculated. Thirdly, we scale it up to the population level to estimate the impact of fully-funded vaccination program on the health and economic outcomes. Methods are shown below in details.

1. National estimates

For the key model parameters as influenza-like-illness (ILI) consultations rate due to influenza and influenza-related excess mortality rate attributable to respiratory diseases, national-level data are available (Supplementary Materials 2, table S1). Accordingly,

$$\mathrm{ICER}_{Nation} = \frac{\sum_{i=1}^{5} Cost\ per\ person\ of\ fully-funded\ vaccination\ program_i - \sum_{i=1}^{5} Cost\ per\ person\ of\ self-paid\ vaccination\ program_i}{\sum_{i=1}^{5} Health\ utility\ loss\ per\ person\ of\ of\ self-paid\ vaccination\ program_i - \sum_{i=1}^{5} Health\ utility\ loss\ per\ person\ fully-funded\ vaccination\ program_i}$$

Where i denotes age groups, including 60-, 65-, 70-, 75-, and 80+ years old.

$$National\ cost\ of\ fully-funded\ vaccination\ program\ at\ the\ population\ level = \sum_{i=1}^{5} Cost\ per\ person\ of\ fully-funded\ vaccination\ program_i \times national\ population\ size_i$$

Other national health and economic outcomes at the population level were calculated in the same way.

2. Regional estimates

With regards to the influenza-related ILI consultation and excess mortality rates, although regional-level data are unavailable, for at least two provinces in each geographic region, the data are available (Supplementary Materials 2, table S1) and thus these provinces were included in the analysis. We calculated the overall ICER for each region by weighting the provincial ICERs using the proportion of age-specific population in total population size of older adults of these provinces which were included in the analysis.

$$\mathrm{ICER}_{Region}=\frac{\sum_{i=1}^{5}\sum_{p=1}^{k}Cost\ per\ person\ of\ fully{-}funded\ vaccination\ program_{ip}\times\frac{Provincial\ population\ size_{pi}}{\sum_{i=1}^{5}\sum_{p=1}^{k}Provincial\ population\ size_{ip}}-\sum_{i=1}^{5}\sum_{p=1}^{k}Cost\ per\ person\ of\ self{-}paid\ vaccination\ program_{ip}\times\frac{Provincial\ population\ size_{ip}}{\sum_{i=1}^{5}\sum_{p=1}^{k}Provincial\ population\ size_{ip}}}{\sum_{i=1}^{5}\sum_{p=1}^{k}Helath\ utility\ loss\ per\ person\ of\ self{-}paid\ vaccination\ program_{ip}\times\frac{Provincial\ population\ size_{pi}}{\sum_{i=1}^{5}\sum_{p=1}^{k}Provincial\ population\ size_{ip}}-\sum_{i=1}^{5}\sum_{p=1}^{k}Helath\ utility\ loss\ per\ person\ of\ fully{-}funded\ vaccination\ program_{ip}\times\frac{Provincial\ population\ size_{ip}}{\sum_{i=1}^{5}\sum_{p=1}^{k}Provincial\ population\ size_{ip}}}$$

Where i denotes age groups, including 60-, 65-, 70-, 75-, and 80+ years old, p denotes provinces with influenza-related burden available, and k denotes the number of provinces in a specific region.

$$Regional\ cost\ of\ fully-funded\ vaccination\ program\ at\ the\ population\ level=\sum_{i=1}^{5}\sum_{p=1}^{k}Cost\ per\ person\ of\ fully-funded\ vaccination\ program_{ip}\times\frac{Provincial\ population\ size_{ip}}{\sum_{i=1}^{5}\sum_{p=1}^{k}Provincial\ population\ size_{ip}+}\times Regional\ population\ size\ of\ older\ adults$$

Other regional health and economic outcomes at the population level were calculated in the same way.
