# Supplementary material for: Cost-effectiveness of introducing national seasonal influenza vaccination for adults aged 60 years and above in mainland China: a modelling analysis
Source: BMC Med. 2020 Apr 14;18:90. doi: 10.1186/s12916-020-01545-6 (PMC7155276; doi:10.1186/s12916-020-01545-6)
Supplement: Supplementary file 2 — Figure S1. Map showing geographical regions in mainland China. [file 12916_2020_1545_MOESM2_ESM.pdf]

Additional file 2. Map of geographic regions, mainland China

Mainland China are officially split into two and seven geographic regions as follows:

1. Two geographic regions
   - Northern: Beijing, Tianjin, Liaoning, Jilin, Heilongjiang, Shandong, Henan, Shaanxi, Gansu and Tibet
   - Southern: Jiangsu, Zhejiang, Anhui, Fujian, Hubei, Hunan, Guangdong, Guangxi, Chongqing, Sichuan, and Guizhou
2. Seven geographic regions
   - Northern: Beijing, Tianjin, Hebei, Shanxi, and Inner Mongolia
   - Northeast: Liaoning, Jilin, and Heilongjiang
   - Eastern: Shanghai, Jiangsu, Zhejiang, Anhui, Fujian, Jiangxi and Shandong
   - Central: Henan, Hubei and Hunan
   - Southern: Guangdong, Guangxi and Hainan
   - Southwest: Chongqing, Sichuan, Guizhou, Yunnan and Tibet
   - Northwest: Shaanxi, Gansu, Qinghai, Ningxia and Xinjiang

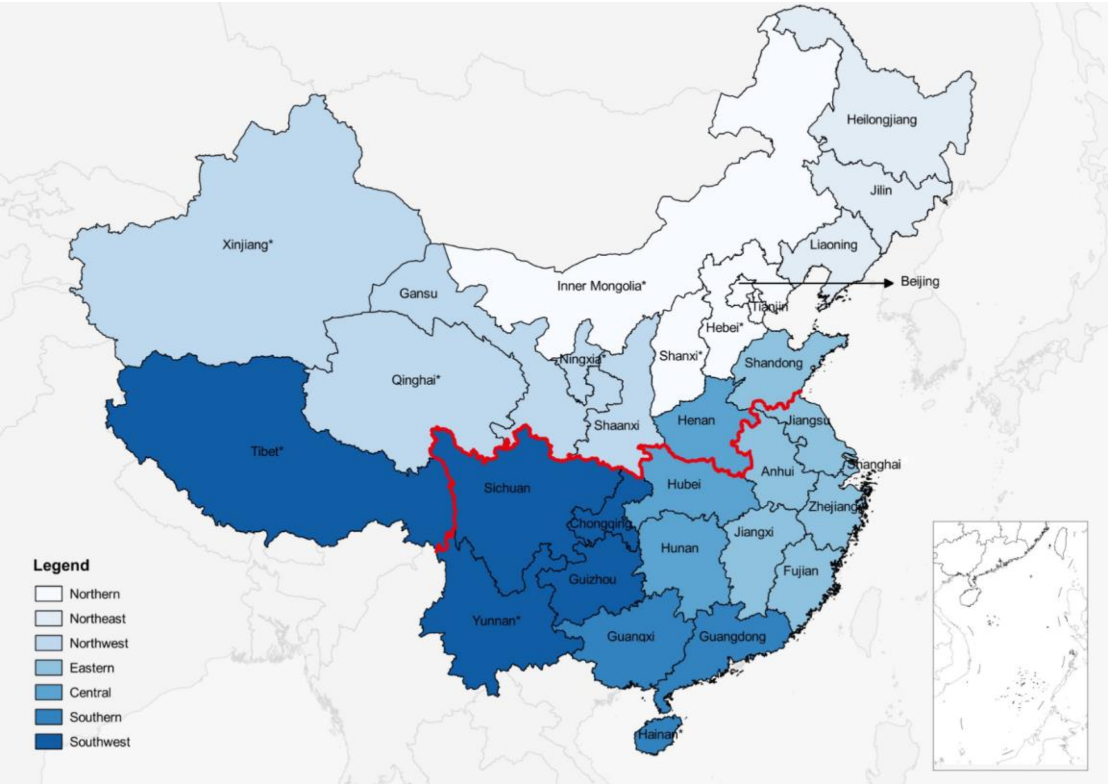

Figure S1. Map showing the seven geographical regions in mainland China (*denote the provinces excluded in our analysis due to unavailability of influenza-related respiratory excess mortality estimates based on local surveillance data, including Hebei, Shanxi, Inner Mongolia, Hainan, Yunnan, Tibet, Qinghai, Ningxia and Xinjiang) (with Southern/Northern stratification, the red line separates the Northern and Southern provinces)
