# Supplementary material for: Cost-effectiveness of introducing national seasonal influenza vaccination for adults aged 60 years and above in mainland China: a modelling analysis
Source: BMC Med. 2020 Apr 14;18:90. doi: 10.1186/s12916-020-01545-6 (PMC7155276; doi:10.1186/s12916-020-01545-6)
Supplement: Supplementary file 3 — Table S1. Population size. [file 12916_2020_1545_MOESM3_ESM.pdf]

Additional file 3. Population size

Table S1. Age-specific population size in 2016 and proportion of urban residents in the elderly aged 60 years and over, China

Region	Number of elderly population in 2016						Number of elderly population in 2010 Census [24]		
	60-	65-	70-	75-	≥80	Total	Urban areas*	Total	Proportion of population in urban areas (%)
**Northern**†	8,709,204	5,501,341	4,469,037	3,586,402	2,980,987	25,246,971	/	/	/
Beijing	887,061	618,763	631,518	547,562	432,622	3,117,526	2,056,206	2,460,108	83.6
Tianjin	799,155	504,610	387,915	332,246	310,829	2,334,755	1,303,592	1,684,685	77.4
Subtotal‡	1,686,216	1,123,373	1,019,433	879,808	743,451	5,452,281	3,359,798	4,144,793	81.1
**Northeast**†	6,291,490	4,086,626	3,412,485	2,434,404	2,120,108	18,345,113	8,995,198	15,369,915	58.5
Liaoning	2,546,440	1,793,694	1,454,519	1,103,511	1,028,502	7,926,666	4,066,177	6,750,752	60.2
Jilin	1,600,564	976,675	802,018	550,725	474,940	4,404,922	1,988,854	3,626,548	54.8
Heilongjiang	2,144,486	1,316,257	1,155,948	780,168	616,666	6,013,525	2,940,167	4,992,615	58.9
Subtotal‡	6,291,490	4,086,626	3,412,485	2,434,404	2,120,108	18,345,113	8,995,198	15,369,915	58.5
**Northwest**†	4,138,090	3,576,331	2,843,240	1,911,816	1,200,930	13,670,407	/	/	/
Shaanxi	1,741,193	1,452,145	1,127,806	791,829	546,443	5,659,416	1,930,406	4,796,819	40.2
Gansu	1,115,233	988,253	783,252	555,025	281,301	3,723,064	1,005,469	3,180,858	31.6
Subtotal‡	2,856,426	2,440,398	1,911,058	1,346,854	827,744	9,382,480	2,935,875	7,977,677	36.8
**Eastern**†	20,841,073	15,171,295	11,067,830	9,597,123	9,559,848	66,237,169	/	/	/
Shanghai	1,314,373	822,634	540,230	672,949	715,392	4,065,578	3,037,617	3,469,655	87.5
Jiangsu	4,509,526	3,424,665	2,518,335	2,071,300	2,109,054	14,632,880	6,415,633	12,574,637	51.0
Zhejiang	2,945,848	1,888,489	1,392,201	1,399,713	1,391,743	9,017,994	3,615,547	7,558,633	47.8
Anhui	3,092,465	2,689,781	1,731,704	1,472,544	1,399,248	10,385,742	3,209,560	8,931,533	35.9
Fujian	1,483,011	1,174,122	985,834	782,993	843,944	5,269,904	1,976,740	4,212,388	46.9
Jiangxi	2,034,514	1,360,336	1,126,435	854,986	763,388	6,139,659	2,051,178	5,099,258	40.2
Shandong	5,461,336	3,811,268	2,773,091	2,342,638	2,337,079	16,725,412	5,890,238	14,130,461	41.7

Subtotal‡	20,841,073	15,171,295	11,067,830	9,597,123	9,559,848	66,237,169	26,196,513	55,976,565	46.8
**Central**†	11,468,304	8,228,294	5,837,144	4,894,154	4,282,840	34,710,736	10,919,892	29,498,021	37.0
Henan	4,710,130	3,382,826	2,206,422	1,892,366	1,752,700	13,944,444	3,949,435	11,968,210	33.0
Hubei	3,152,382	2,366,137	1,533,074	1,375,022	1,064,841	9,491,456	3,440,366	7,973,958	43.1
Hunan	3,605,792	2,479,331	2,097,648	1,626,766	1,465,299	11,274,836	3,530,091	9,555,853	36.9
Subtotal‡	11,468,304	8,228,294	5,837,144	4,894,154	4,282,840	34,710,736	10,919,892	29,498,021	37.0
**Southern**†	5,883,760	4,401,043	3,729,775	3,083,410	3,102,131	20,200,119	/	/	/
Guangdong	3,554,752	2,513,452	2,139,881	1,824,274	1,885,929	11,918,288	5,626,058	10,152,391	55.4
Guangxi	1,991,489	1,670,878	1,360,867	1,069,113	1,020,148	7,112,495	2,026,426	6,036,234	33.6
Subtotal‡	5,546,241	4,184,330	3,500,748	2,893,387	2,906,077	19,030,783	7,652,484	16,188,625	47.3
**Southwest**†	9,678,523	8,450,148	6,285,883	4,250,756	4,112,467	32,777,777	/	/	/
Chongqing	1,741,535	1,578,106	1,059,381	760,486	735,970	5,875,478	2,117,648	5,024,394	42.1
Sichuan	4,538,640	3,995,193	2,868,499	1,858,501	1,928,087	15,188,920	4,367,383	13,109,909	33.3
Guizhou	1,571,294	1,344,603	1,117,797	744,128	617,860	5,395,682	1,242,446	4,461,272	27.8
Subtotal‡	7,851,469	6,917,902	5,045,677	3,363,115	3,281,917	26,460,080	7,727,477	22,595,575	34.2
**Nation**	**67,010,444**	**49,415,078**	**37,645,394**	**29,758,065**	**27,359,311**	**211,188,292**	**78,291,143**	**177,594,440**	**44.1**

* Including the population in city and township in the 2010 census.
† Total population for the region.
‡ Total population for the provinces of the region which are included in the study (Supplementary Materials 2, figure S1).
/ not used in the study.
