# Supplementary material for: Cost-effectiveness of introducing national seasonal influenza vaccination for adults aged 60 years and above in mainland China: a modelling analysis
Source: BMC Med. 2020 Apr 14;18:90. doi: 10.1186/s12916-020-01545-6 (PMC7155276; doi:10.1186/s12916-020-01545-6)
Supplement: Supplementary file 4 — Figure S2. Schematic diagram for estimating high-risk population size; Figure S3. Probability of having underlying diseases. [file 12916_2020_1545_MOESM4_ESM.pdf]

Additional file 4. Estimating the proportion of adults aged 60 years and over in high risk groups

High risk groups refer to individuals with underlying medical conditions who are at increased risk of hospitalization or death if infected by influenza as listed in the WHO guidelines, including chronic obstructive pulmonary disease, asthma, diabetes, chronic cardiac disease, chronic renal disease, chronic liver disease, chronic neurological disease, chronic haematological disorder, obesity and tuberculosis, etc.[25] In China, the probability of having at least one underlying medical disease in the older adults is unclear. We aim to work out the proportion of high risk groups in adults aged 60 years and above using the open dataset of "China Health and Retirement Longitudinal Study". [26]

The "China Health and Retirement Longitudinal Study" is a nationally representative study on social, economic and health status (including self-reported doctor-diagnosed chronic diseases) in the persons aged 45 years of age and older in China. The national baseline survey was performed in 2011 and then followed up in 2013. The dataset in 2013 included the baseline survey and follow-up survey results for participants enrolled in 2011, as well as the baseline survey results for new enrolled participants in 2013. Details with regarding to the CHARLS were presented in previous publications.[27] We retrieved the demographics and baseline self-reported chronic diseases for the old persons aged 60 years and over from the 2013 datasets.

Excluding 79 respondents without age information, the baseline survey involved 7416 older respondents. Excluding 84 respondents with all interested chronic diseases information unavailable, 7,332 old participants were included in our analysis. We used World Health Organization definition of obesity as a body mass index (BMI) of >30 kg/m^2, and calculated it on the basis of height and weight, which was measured at the point of questionnaire interviews. 27.2% (n=1,994/7,332) of participants refused to take anthropometric measurements. The details regarding missing data process and calculating the probability of having ⩾1 underlying chronic disease (including diabetes, chronic lung

diseases, chronic liver diseases, chronic heart diseases, stroke, chronic kidney diseases, asthma, and obesity) were presented in figure S2 and below.

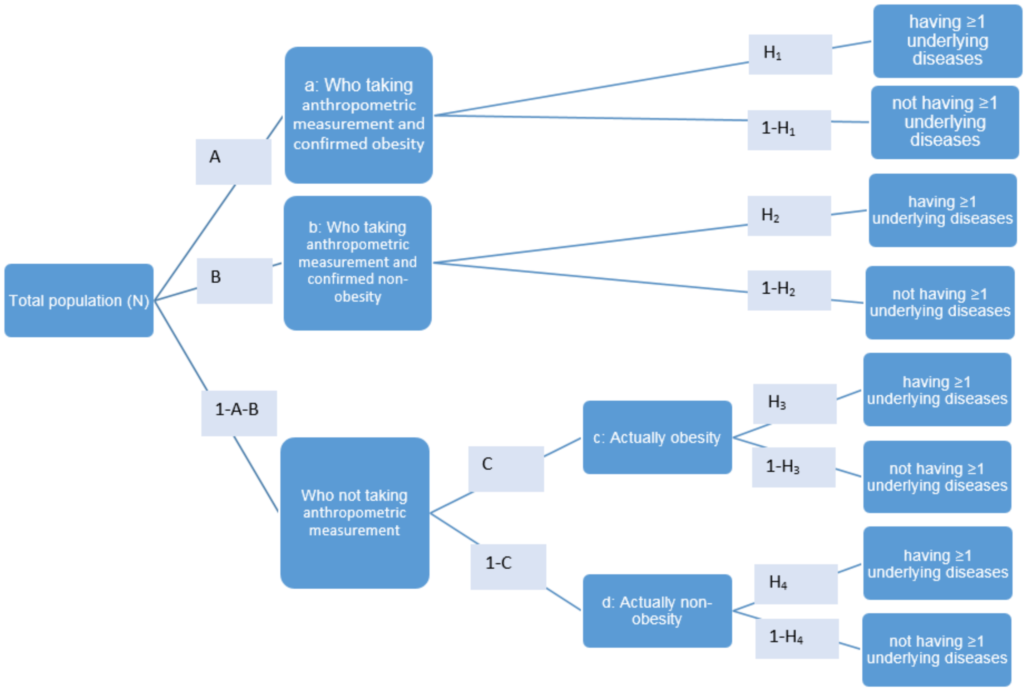

Figure S2. Schematic diagram

Denotations in figure S2:

A: the proportion of those taking anthropometric measurement and confirmed obesity;

B: the proportion of those taking anthropometric measurement and confirmed non-obesity;

C: the proportion of obesity in those not taking anthropometric measurement;

Hn: the proportion of having ≥1 underlying diseases separately in population a, b, c and d.

We assumed that,

1) In those not taking anthropometric measurement and actually having obesity, the probability of having ≥1 underlying diseases is same with

those taking anthropometric measurement and confirmed having obesity, i.e., $H_3=H_1$

2) In those not taking anthropometric measurement and actually not having obesity, the probability of having ≥1 underlying diseases is same with those taking anthropometric measurement and confirmed not having obesity, i.e., $H_4=H_2$

3) The probability of taking anthropometric measurement in persons not having obesity (denoted as P_{t1}) is R times that in persons having obesity (denoted as P_{t2}), i.e.,

$P_{t2}=R^* P_{t1}$

(1) $P_{t1} = \frac{A}{A+(1-A-B)*C}$

(2) $P_{t2} = \frac{B}{B+(1-A-B)*(1-C)}$

(3)

Based on (1)-(3),

$$C = \frac{A*(R-R*A-B)}{(B+R*A)*(1-A-B)}$$

(4)

Therefore, the probability of having at least one chronic disease in the total population (denoted as $P_{(HR)}$) is

$$P_{HR} = \mathrm{A} * \mathrm{H}_1 + \mathrm{B} * \mathrm{H}_2 + (1 - \mathrm{A} - \mathrm{B}) * \mathrm{C} * \mathrm{H}_1 + (1 - \mathrm{A} - \mathrm{B}) * (1 - \mathrm{C}) * \mathrm{H}_2$$

(5)

Because $H_1=1$,

$$P_{HR} = \mathrm{A} + \mathrm{B} * \mathrm{H}_2 + [(1 - (\mathrm{A} + \mathrm{B})] * [\mathrm{C} + (1 - \mathrm{C}) * \mathrm{H}_2]$$

(6)

Accordingly, if R=1 which means persons with obesity have same probability of refusing anthropometric measurements with those without obesity,

$$C = \frac{A}{A+B}$$
(7)

Substitute (7) into (6), we then get

$$P_{HR} = \frac{A+B*H_2}{A+B}$$

We estimated the mean and 95%CI of the probability of having at least one underlying chronic disease using Beta distribution. Fig S3 showed the results for the elderly when the probability of taking anthropometric measurement in persons not having obesity is the same with that in persons having obesity.

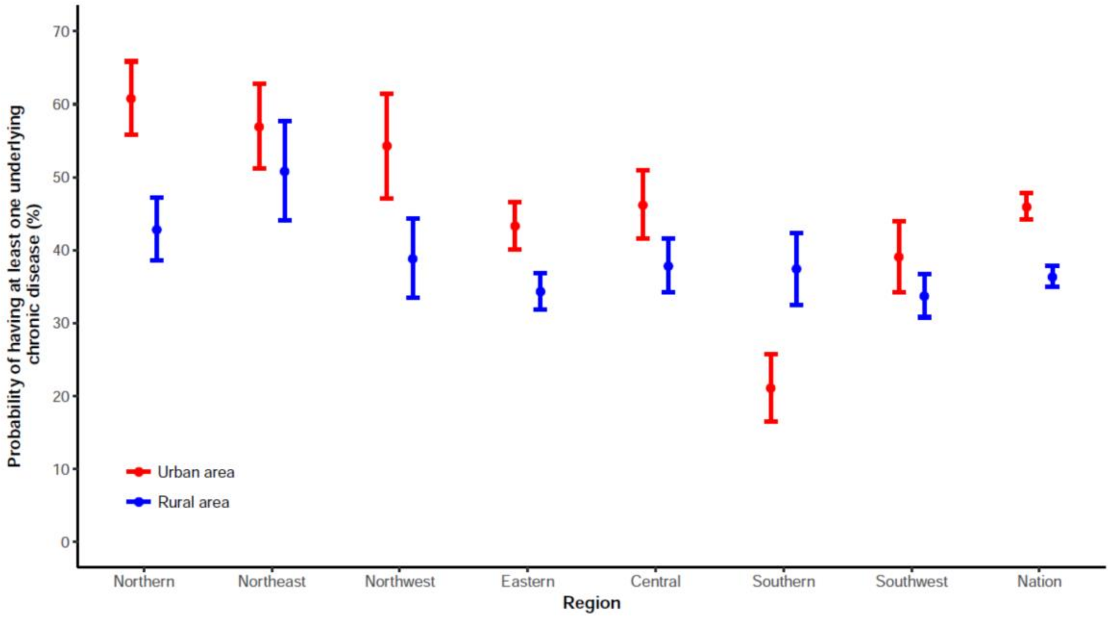

Figure S3. The probability of having ≥1 underlying chronic disease in persons aged 60 years and over (%) (mean, 95% confidence interval)
