# Supplementary material for: Cost-effectiveness of introducing national seasonal influenza vaccination for adults aged 60 years and above in mainland China: a modelling analysis
Source: BMC Med. 2020 Apr 14;18:90. doi: 10.1186/s12916-020-01545-6 (PMC7155276; doi:10.1186/s12916-020-01545-6)
Supplement: Supplementary file 5 — Table S2. Influenza-related disease burden. [file 12916_2020_1545_MOESM5_ESM.pdf]

Additional file 5. Influenza-related disease burden in older adults, China

Table S2. Influenza-like-illness (ILI) consultations due to influenza and influenza-related respiratory excess mortality for elderly aged 60 or above, stratified by provinces in China between 2010-2015 (mean, 95%CI)

Region	Province	Influenza-associated ILI consultations (per 100,000 population)[28]	Influenza-related respiratory excess mortality (per 100,000 population)[12]
Northern	Beijing	470 (360, 580)	72.7 (64.6, 81.2)
	Tianjin	480 (320, 650)	18.8 (13.5, 23.5)
Northeast	Liaoning	60 (40, 80)	23.0 (20.1, 27.2)
	Jilin	10 (-10, 30)	19.3 (16.3, 22.4)
	Heilongjiang	60 (20, 100)	19.8 (16.5, 23.1)
Northwest	Shaanxi	20 (-10, 50)	38.9 (34.0, 44.0)
	Gansu	20 (0, 30)	83.2 (70.8, 93.9)
Eastern	Shanghai	690 (530, 870)	37.8 (31.5, 43.9)
	Jiangsu	80 (40, 130)	48.0 (42.0, 54.2)
	Zhejiang	230 (170, 290)	57.3 (52.8, 61.7)
	Anhui	70 (40, 90)	29.9 (25.0, 34.0)
	Fujian	60 (30, 90)	63.1 (53.4, 72.2)
	Jiangxi	80 (60, 100)	12.5 (1.3, 24.8)
	Shandong	30 (10, 50)	41.0 (37.2, 46.5)
Central	Henan	20 (-10, 60)	19.0 (15.5, 22.6)
	Hubei	130 (70, 180)	20.2 (16.3, 23.9)
	Hunan	20 (10, 40)	42.0 (35.3, 50.7)
Southern	Guangdong	180 (110, 260)	34.9 (23.4, 48.5)
	Guangxi	40 (30, 60)	63.9 (48.8, 76.9)
Southwest	Chongqing	10 (10, 20)	44.0 (36.4, 52.0)
	Sichuan	10 (0, 20)	27.3 (20.3, 38.0)
	Guizhou	10 (0, 30)	55.1 (41.6, 69.8)
Nation	Nation	90 (40, 150)	38.5 (36.8, 40.2)
