# Supplementary material for: Cost-effectiveness of introducing national seasonal influenza vaccination for adults aged 60 years and above in mainland China: a modelling analysis
Source: BMC Med. 2020 Apr 14;18:90. doi: 10.1186/s12916-020-01545-6 (PMC7155276; doi:10.1186/s12916-020-01545-6)
Supplement: Supplementary file 6 — Table S3. Age-specific mortality by rural/urban areas; Table S4. Life expectancy. [file 12916_2020_1545_MOESM6_ESM.pdf]

Additional file 6. Age-specific life expectancy in the older adults, China

We figured out the risk- (HRs and LRs mentioned in Supplementary Materials 3) and age-specific life expectancy stratified by urban/rural areas by assuming that all mortality due to high risk diseases occurs in HRs, while mortality due to other causes (i.e., other than high risk diseases) occurs equally in HRs and LRs. Therefore, the mortality in LRs equals to all-cause mortality minus HRs disease related mortality. HRs population may die from HRs diseases or other causes. So,

$$\text{The mortality of HRs population} = \frac{\text{the number of HRs population death}}{\text{the number of HRs population}} = \frac{\text{the number of HRs population death}}{\text{total population size} \times \text{HRs proportion}} = \frac{\text{HRs disease related mortality}}{\text{HRs proportion}} + (\text{all cause mortality} - \text{HRs disease related mortality})$$

(8)

The age-specific mortality in 2017 was presented in table S3. The HRs proportion stratified by rural/urban areas was presented in Fig S3. The life expectancy in LRs is not affected by HRs proportion. The life table approach used in estimating the life expectancy on the basis of risk-specific mortality were described in “WHO methods for life expectancy and healthy life expectancy”.[39] (Table S4)

Table S3. Age-specific mortality stratified by rural/urban areas in the elderly in China in 2017 (1/100,000).[31]

	60-	65-	70-	75-	80-	≥85
Urban area						
All diseases	1020.81	1616.34	2223.63	3518.55	7601.16	19231.9
High risk diseases*						
Chronic lower respiratory disease	42.19	88.51	173.57	328.16	789.56	2116.37
Diabetes	31.28	54.95	79.58	120.52	247.87	448.59
Hypertensive heart disease	13.49	27.2	45.33	86.71	238.79	772.67
Coronary heart disease	136.3	235.43	359.55	662.37	1683.37	5103.43
Congenital heart disease	0.35	0.27	0.33	0.31	0.39	0.74
Chronic renal failure	4.18	6.57	8.67	11.52	22.07	47.68
Hepatic disease	15.31	18.81	17.41	22.28	38.59	67.26
Stroke	4.45	9.39	16.85	28.61	67.23	183.97
Hematologic disease	1.48	3.22	3.93	6.14	14.64	33.11
AIDS	1.13	0.76	0.7	0.81	1.17	1.03
Respiratory tract tuberculosis	3.41	4.56	5	6.99	12.68	20.75
Total	253.57	449.67	710.92	1274.42	3116.36	8795.60
Rural area						
All diseases	1067.61	1749.23	2689.76	4120.11	7368.13	16683.92
High risk diseases*						
Chronic lower respiratory disease	51.82	115.13	257.07	496.87	1039.72	2493.81
Diabetes	26.26	49.32	74.69	100.09	141.99	205.13
Hypertensive heart disease	15.11	32.65	67.16	128.55	302.88	820.61
Coronary heart disease	148.66	264.28	450.14	768.43	1588.43	4247.27
Congenital heart disease	0.18	0.14	0.11	0.09	0.07	0.55
Chronic renal failure	3.36	4.83	6.62	9.31	13.12	23.38
Hepatic disease	13.85	16.86	19.31	22.63	25.75	34.62
Stroke	5.93	11.88	23.7	45.62	91.5	231.98
Hematologic disease	1.77	2.74	4.18	5.57	10.53	23.59
AIDS	0.84	1.05	0.66	0.78	0.6	0.49
Respiratory tract tuberculosis	4.32	6.97	9.1	12.51	14.43	16.44
Total	272.10	505.85	912.74	1590.45	3229.02	8097.87

*High risk disease refers to those could increase risk of hospitalization or death if infected by influenza as listed in the WHO guidelines.

Table S4. Average age-specific life expectancy in the elderly in China

Region	Area	60-		65-		70-		75-		≥80	
		HRs	LRs	HRs	LRs	HRs	LRs	HRs	LRs	HRs	LRs
Northern	Urban	19·18	21·32	15·19	17·06	11·46	12·93	7·75	8·75	4·03	4·50
	Rural	17·81	21·07	13·95	16·82	10·43	12·74	7·15	8·69	3·87	4·53
Northeast	Urban	19·04	21·32	15·08	17·06	11·37	12·93	7·69	8·75	4·00	4·50
	Rural	18·25	21·07	14·33	16·82	10·74	12·74	7·36	8·69	3·96	4·53
Northwest	Urban	18·94	21·32	14·99	17·06	11·30	12·93	7·64	8·75	3·98	4·50
	Rural	17·53	21·07	13·71	16·82	10·23	12·74	7·01	8·69	3·81	4·53
Eastern	Urban	18·44	21·32	14·55	17·06	10·96	12·93	7·41	8·75	3·87	4·50
	Rural	17·18	21·07	13·39	16·82	9·98	12·74	6·84	8·69	3·73	4·53
Central	Urban	18·44	21·32	14·55	17·06	10·96	12·93	7·41	8·75	3·87	4·50
	Rural	17·18	21·07	13·39	16·82	9·98	12·74	6·84	8·69	3·73	4·53
Southern	Urban	16·18	21·32	12·60	17·06	9·42	12·93	6·35	8·75	3·37	4·50
	Rural	17·43	21·07	13·61	16·82	10·15	12·74	6·96	8·69	3·79	4·53
Southwest	Urban	18·17	21·32	14·32	17·06	10·78	12·93	7·28	8·75	3·81	4·50
	Rural	17·12	21·07	13·34	16·82	9·94	12·74	6·81	8·69	3·72	4·53
