# Supplementary material for: Cost-effectiveness of introducing national seasonal influenza vaccination for adults aged 60 years and above in mainland China: a modelling analysis
Source: BMC Med. 2020 Apr 14;18:90. doi: 10.1186/s12916-020-01545-6 (PMC7155276; doi:10.1186/s12916-020-01545-6)
Supplement: Supplementary file 7 — Figure S4. Epidemiological and economic impact by regions and risk groups; Figure S5. Monte Carlo simulation results by regions and risk groups. [file 12916_2020_1545_MOESM7_ESM.pdf]

Additional file 7. Additional results for the high-risk and low-risk groups from the societal perspective

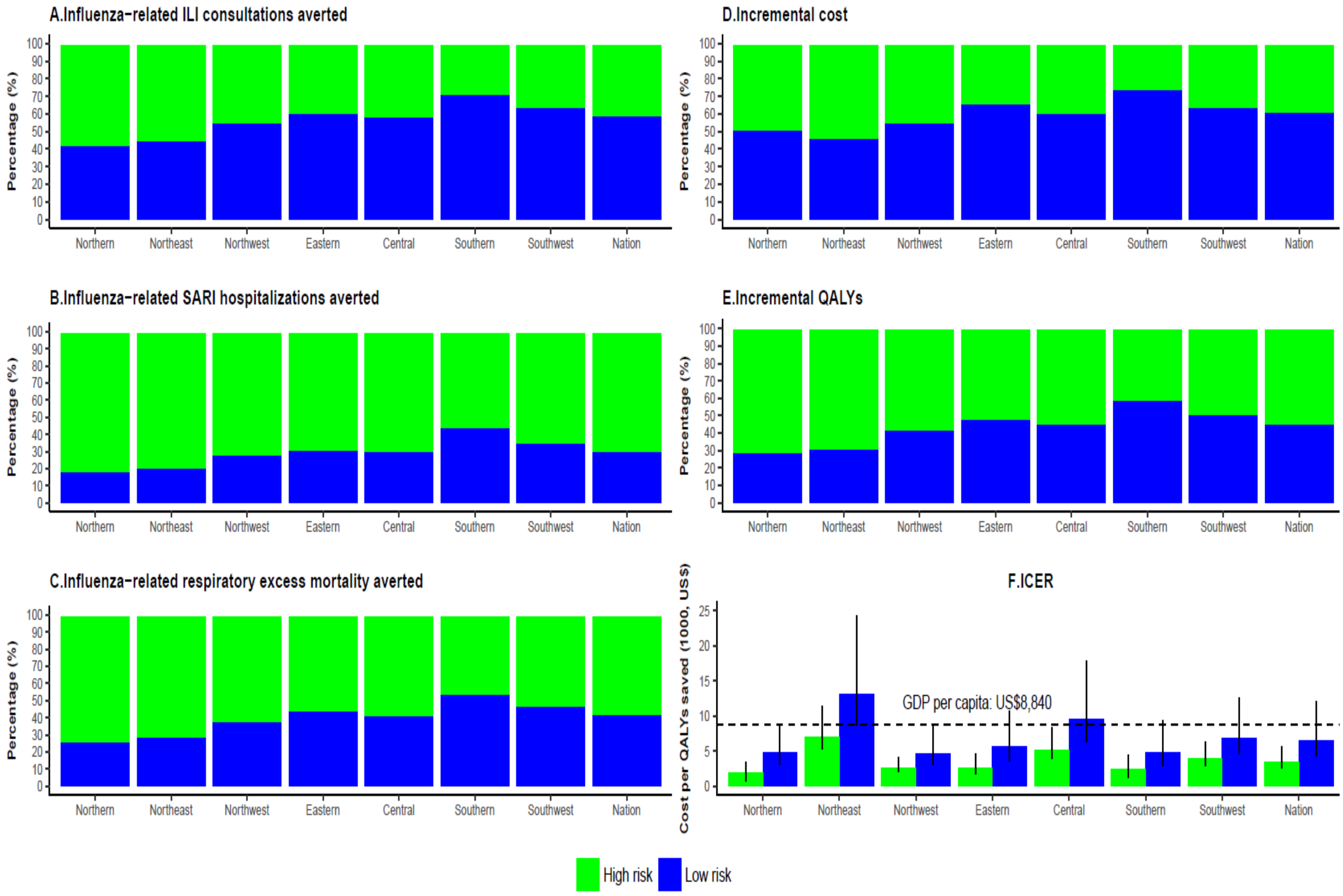

Figure S4. Epidemiological and economic impact of fully-funded influenza vaccination program in older adults, stratified by geographic regions and risk groups, China. "High risk" refers to individuals with underlying medical conditions who are at increased risk of hospitalization or death if infected by influenza as listed in the WHO guidelines, including chronic obstructive pulmonary disease, asthma, diabetes, chronic cardiac disease, chronic renal disease, chronic liver disease, chronic neurological disease, chronic haematological disorder, obesity and tuberculosis, etc.[25] The remaining population was categorized as "low risk".

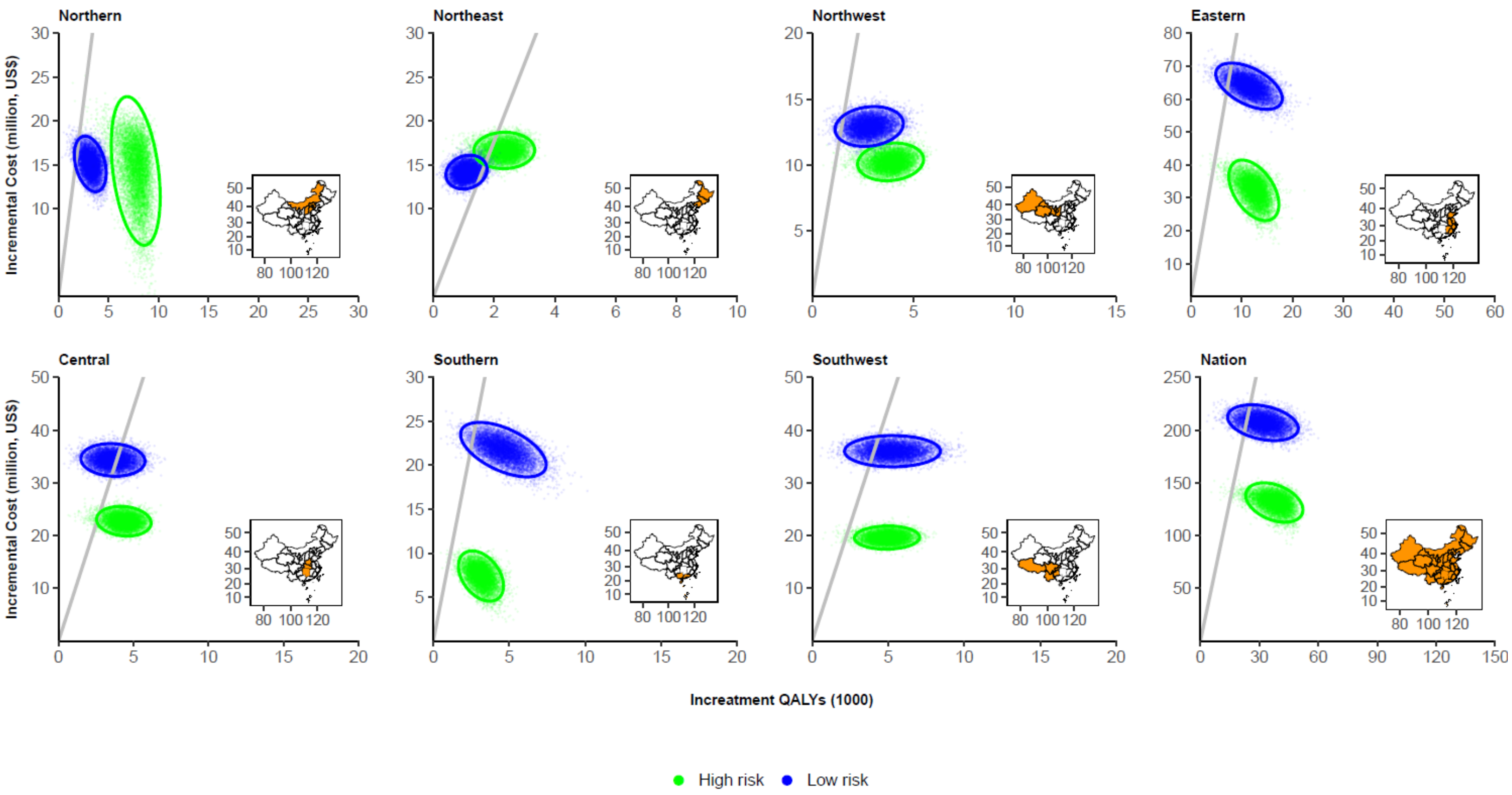

Figure S5. Monte Carlo simulation results on the cost-effectiveness for fully-funded vaccination program compared to self-paid vaccination program, stratified by geographic regions and risk groups, China. "High risk" refers to individuals with underlying medical conditions who are at increased risk of hospitalization or death if infected by influenza as listed in the WHO guidelines, including chronic obstructive pulmonary disease, asthma, diabetes, chronic cardiac disease, chronic renal disease, chronic liver disease, chronic neurological disease, chronic haematological disorder, obesity and tuberculosis, etc.[25] The remaining population was categorized as "low risk". (grey line denotes China's GDP per capita in 2017 and circle denotes the 95%UI)
