# Supplementary material for: Cost-effectiveness of introducing national seasonal influenza vaccination for adults aged 60 years and above in mainland China: a modelling analysis
Source: BMC Med. 2020 Apr 14;18:90. doi: 10.1186/s12916-020-01545-6 (PMC7155276; doi:10.1186/s12916-020-01545-6)
Supplement: Supplementary file 9 — Figure S9. Epidemiological and economic impact (analyses with well-matched vaccines); Figure S10. Monte Carlo simulation results (analyses with well-matched vaccines); Figure S11. CEAC (analyses with well-matched vaccines). [file 12916_2020_1545_MOESM9_ESM.pdf]

Additional file 9. Additional results for analyses with well-matched vaccines

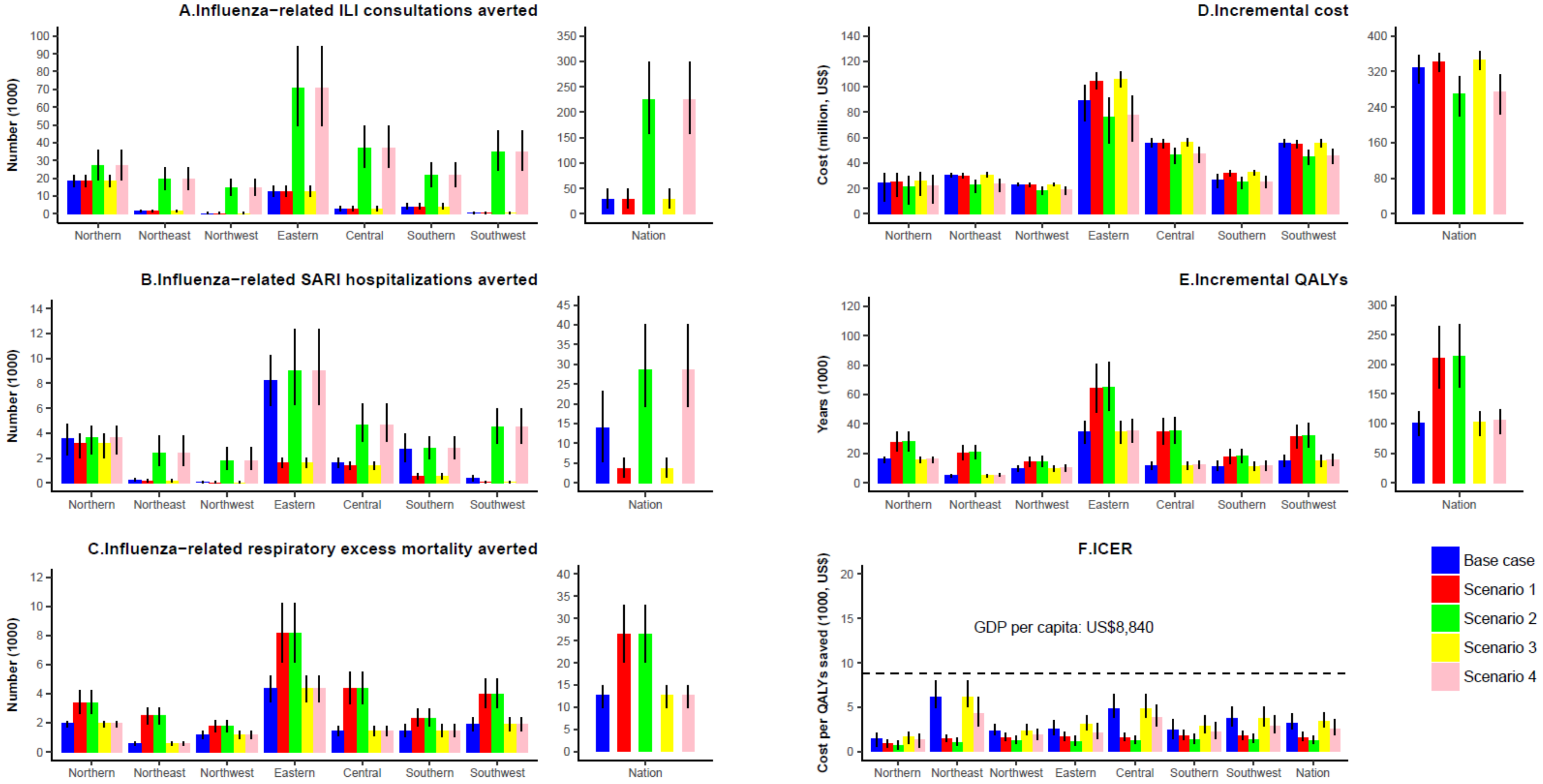

Figure S9. Analyses with well-matched vaccines: Epidemiological and economic impact of fully-funded influenza vaccination program in older adults, stratified by geographic regions, China

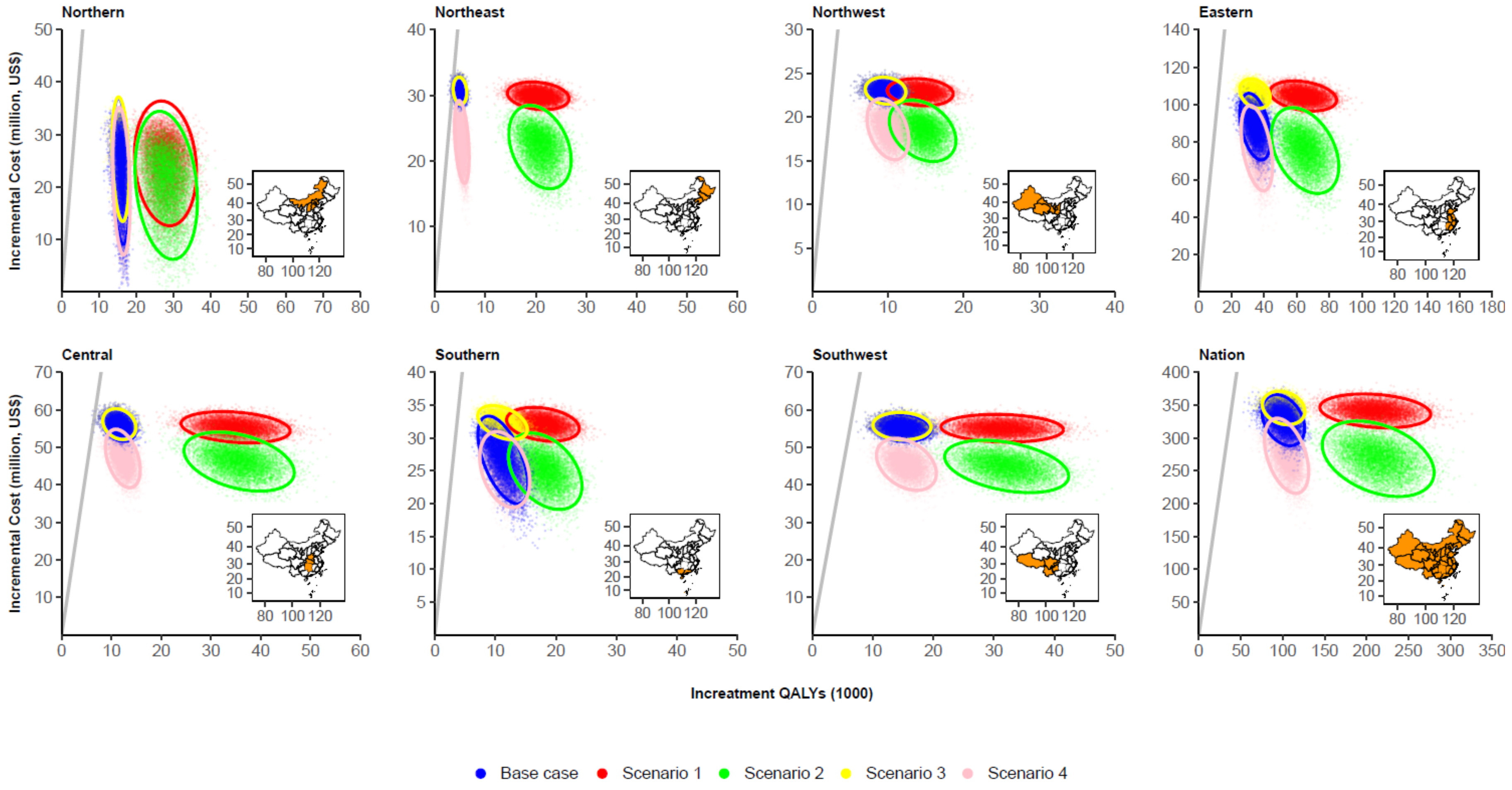

Figure S10. Analyses with well-matched vaccines: Monte Carlo simulation results on the cost-effectiveness for fully-funded vaccination program compared to self-paid vaccination program (grey line denotes China's GDP per capita in 2017 and circle denotes the 95%UI)

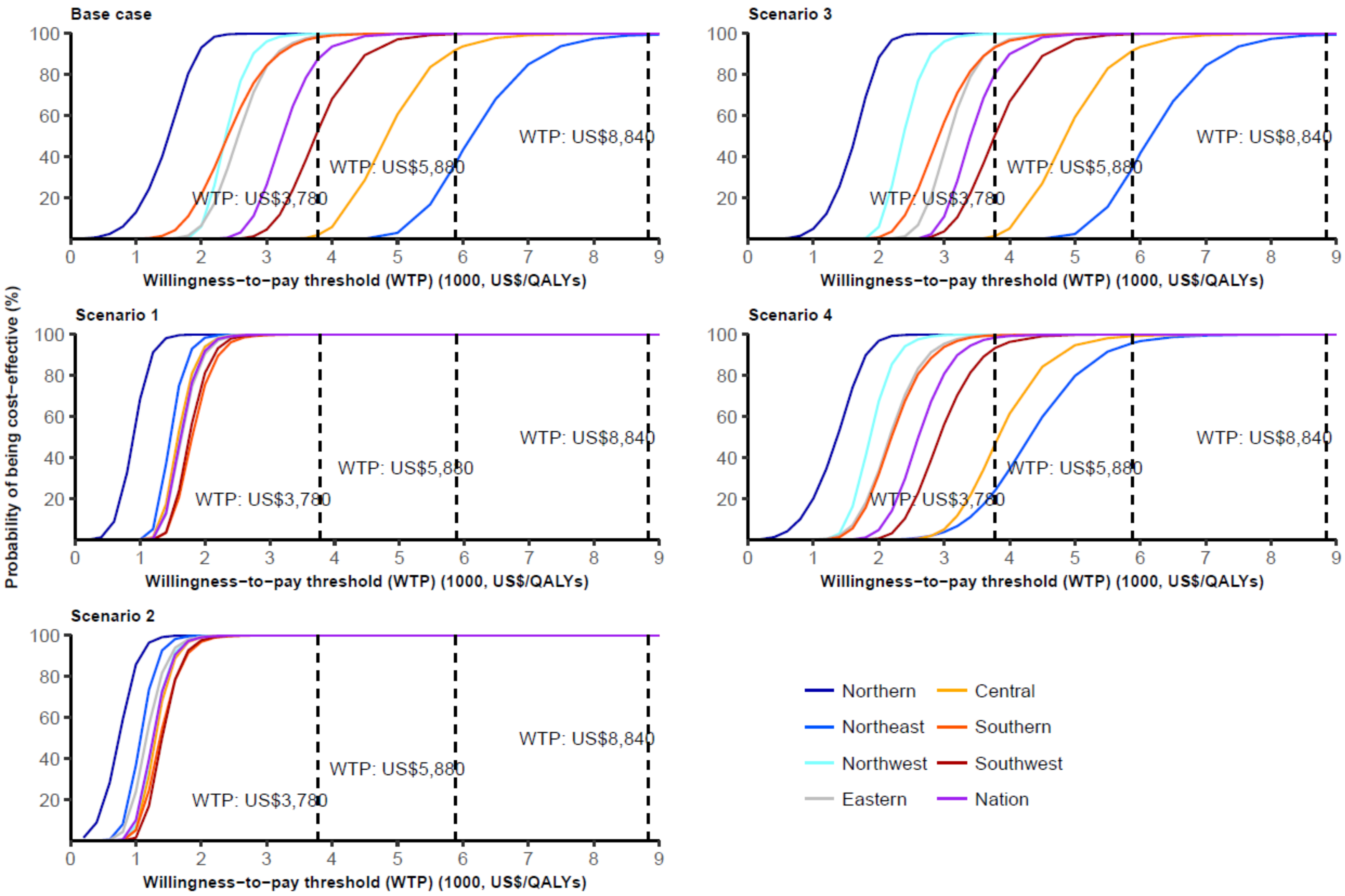

Figure S11. Analyses with well-matched vaccines: Cost-effectiveness acceptability curve (US$3,780 and US$5,880 denote the willingness-to-pay thresholds calculated [47], while US$8,840 is the GDP per capita in 2017, China)
