# Supplementary material for: Cost-effectiveness of introducing national seasonal influenza vaccination for adults aged 60 years and above in mainland China: a modelling analysis
Source: BMC Med. 2020 Apr 14;18:90. doi: 10.1186/s12916-020-01545-6 (PMC7155276; doi:10.1186/s12916-020-01545-6)
Supplement: Supplementary file 11 — Table S5. Comparison of baseline analysis with that using US vaccine effectiveness. [file 12916_2020_1545_MOESM11_ESM.pdf]

Additional file 11. Additional results: Analyses with the vaccine effectiveness between 2013-2018 in the US

Table S5. Comparison of the national epidemiological and economic impact of fully-funded influenza vaccination in baseline analysis using the meta-synthesized vaccine effectiveness[44] with sensitivity analysis using the US vaccine effectiveness*[49] (Median, 95%UI)

	Baseline analysis (vaccine effectiveness=36%)					Sensitivity analysis (vaccine effectiveness=12%)					Sensitivity analysis (vaccine effectiveness=50%)				
	Base case scenario	Scenario 1	Scenario 2	Scenario 3	Scenario 4	Base case scenario	Scenario 1	Scenario 2	Scenario 3	Scenario 4	Base case scenario	Scenario 1	Scenario 2	Scenario 3	Scenario 4
Influenza-related consultations averted	19,812 (7150, 35783)	19,812 (7150, 35783)	155,554 (88896, 226519)	19,812 (7150, 35783)	155,554 (88896, 226519)	6,822 (2667, 10993)	6,822 (2667, 10993)	52,535 (39625, 65726)	6,822 (2667, 10993)	52,535 (39625, 65726)	28,426 (11112, 45805)	28,426 (11112, 45805)	218,896 (165105, 273859)	28,426 (11112, 45805)	218,896 (165105, 273859)
Influenza-related SARI averted	9,418 (3386, 17068)	2,509 (898, 4701)	19,695 (10999, 30122)	2,509 (898, 4701)	19,695 (10999, 30122)	3,247 (1253, 5233)	863 (329, 1448)	6,640 (4790, 8856)	863 (329, 1448)	6,640 (4790, 8856)	13,529 (5219, 21806)	3,598 (1373, 6034)	27,667 (19958, 36899)	3,598 (1373, 6034)	27,667 (19958, 36899)
Influenza-related respiratory excess morality averted	8,800 (5300, 11667)	18,279 (10782, 25433)	18,279 (10782, 25433)	8,800 (5300, 11667)	8,800 (5300, 11667)	2,928 (2836, 3020)	6,115 (5304, 7087)	6,115 (5304, 7087)	2,928 (2836, 3020)	2,928 (2836, 3020)	12,201 (11816, 12583)	25,477 (22101, 29528)	25,477 (22101, 29528)	12,201 (11816, 12583)	12,201 (11816, 12583)
Incremental cost (millions, US$)	339 (310, 363)	348 (328, 369)	299 (256, 335)	351 (331, 371)	302 (259, 338)	355 (336, 374)	358 (339, 377)	341 (321, 362)	359 (340, 378)	342 (322, 363)	329 (297, 356)	342 (322, 362)	273 (227, 309)	346 (326, 367)	277 (231, 313)
Incremental QALYs	70,212 (42106, 93635)	145,356 (85419, 203317)	147,745 (87285, 206175)	70,505 (42226, 93912)	72,830 (43621, 97089)	23,362 (22512, 24228)	48,626 (42176, 56301)	49,429 (42960, 57079)	23,463 (22657, 24285)	24,253 (23295, 25311)	97,341 (93798, 100951)	202,609 (175733, 234587)	205,956 (179000, 237830)	97,762 (94404, 101188)	101,053 (97062, 105463)
ICER	4,832 (3460, 8307)	2,393 (1681, 4146)	2,008 (1313, 3722)	4,986 (3657, 8451)	4,134 (2797, 7534)	15,190 (14140, 16296)	7,362 (6307, 8583)	6,907 (5896, 8067)	15,303 (14343, 16327)	14,114 (13073, 15157)	3,381 (2976, 3747)	1,688 (1438, 1979)	1,320 (1045, 1611)	3,543 (3287, 3816)	2,744 (2258, 3138)

*In the baseline analysis, an average vaccine effectiveness of 36% was obtained from a published meta-analysis of test-negative design case-control studies which were conducted between 2004 and 2013 [44]. In the sensitivity analysis, the most recent vaccine effectiveness between 2013-2018 in the US (range: 12%-50%)[49] were used, with the vaccine effectiveness of 12% and 50% separately for mismatched and well-matched vaccines.
